# Automated Radiosynthesis of *cis*- and *trans*-4-[^18^F]Fluoro-l-proline Using [^18^F]Fluoride

**DOI:** 10.1021/acs.joc.1c00755

**Published:** 2021-04-29

**Authors:** Timaeus E. F. Morgan, Leanne M. Riley, Adriana A. S. Tavares, Andrew Sutherland

**Affiliations:** † BHF-University Centre for Cardiovascular Science, University of Edinburgh, Edinburgh EH16 4TJ, United Kingdom; ‡ WestCHEM, School of Chemistry, University of Glasgow, The Joseph Black Building, Glasgow G12 8QQ, United Kingdom

## Abstract

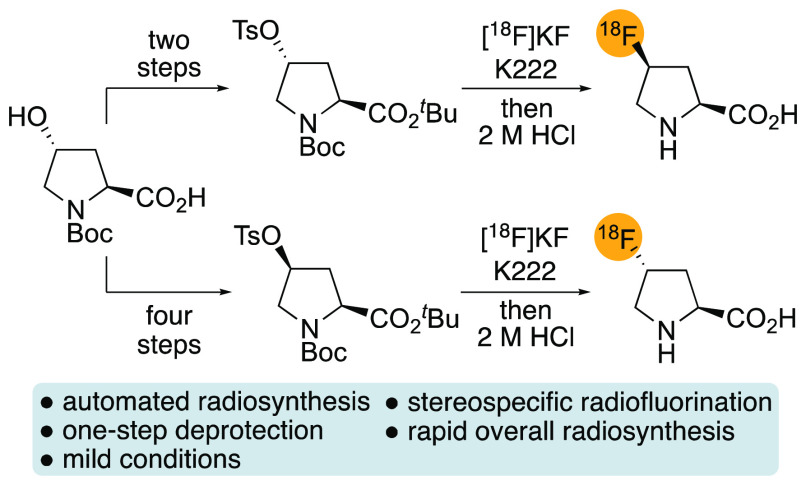

The positron emission tomography imaging agents *cis*- and *trans*-4-[^18^F]fluoro-l-proline are used for the detection of numerous diseases such as pulmonary fibrosis and various carcinomas. These imaging agents are typically prepared by nucleophilic fluorination of 4-hydroxy-l-proline derivatives, with [^18^F]fluoride, followed by deprotection. Although effective radiofluorination reactions have been developed, the overall radiosynthesis process is suboptimal due to deprotection methods that are performed manually, require multiple steps, or involve harsh conditions. Here we describe the development of two synthetic routes that allow access to precursors, which undergo highly selective radiofluorination reactions and rapid deprotection, under mild acidic conditions. These methods were found to be compatible with automation, avoiding manual handling of radioactive intermediates.

## Introduction

**Figure 1:**
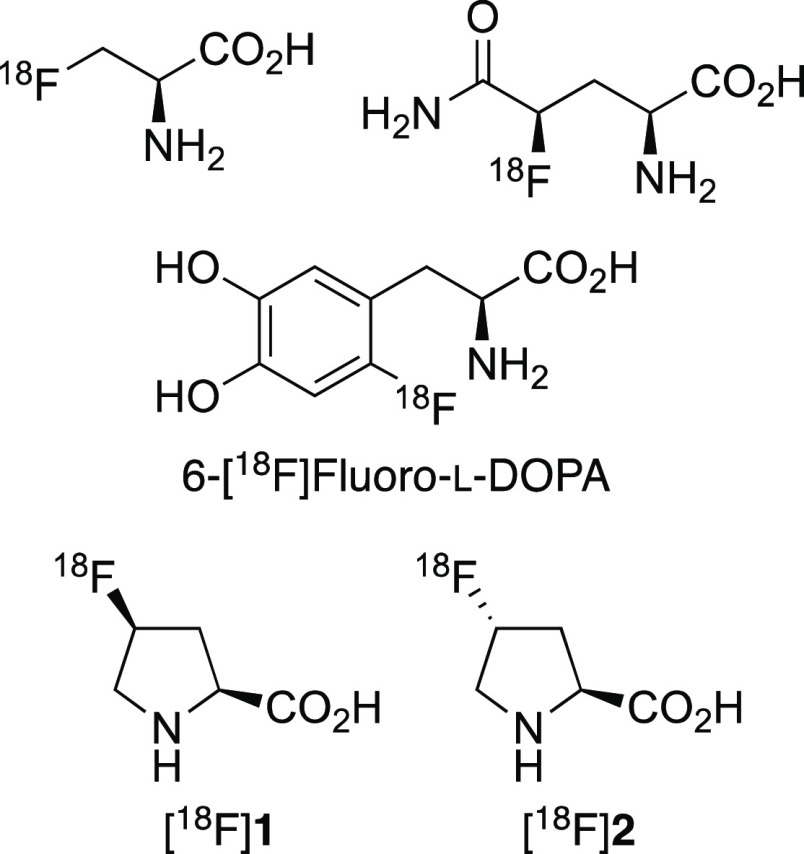
Selected examples of ^18^F-fluorinated α-amino acids.

α-Amino acids are the key building blocks of life, acting as structural components of peptides and proteins.^1^ They also play an important role in biochemical and physiological processes, including energy metabolism, and in the formation of neurotransmitters and hormones. Due to the varied and important roles of α-amino acids in nature, their structural analogues have often been used to study biological processes and mechanisms.^2,3^ Positron emission tomography (PET) in combination with ^18^F-labeled α-amino acids (Figure 1) has been used for the non-invasive generation of molecular, functional, and metabolic information for a wide range of diseases.^4^ Although most applications have focused on imaging various forms of cancer, compounds such as 6-[^18^F]fluoro-l-DOPA have been used to investigate neurodegenerative disorders, including Parkinson’s disease.^5^ The *cis* and *trans* isomers of 4-[^18^F]fluoro-l-proline, [^18^F]**1** and [^18^F]**2**, respectively, have also been used to image a number of disease conditions. Proline and 4-hydroxyproline are major structural components of collagen (15–30%), and therefore, [^18^F]**1** and [^18^F]**2** have been used to investigate abnormal collagen biosynthesis in diseases such as liver cirrhosis, lung fibrosis, and various carcinomas.^4,6,7^

**Scheme 1:**
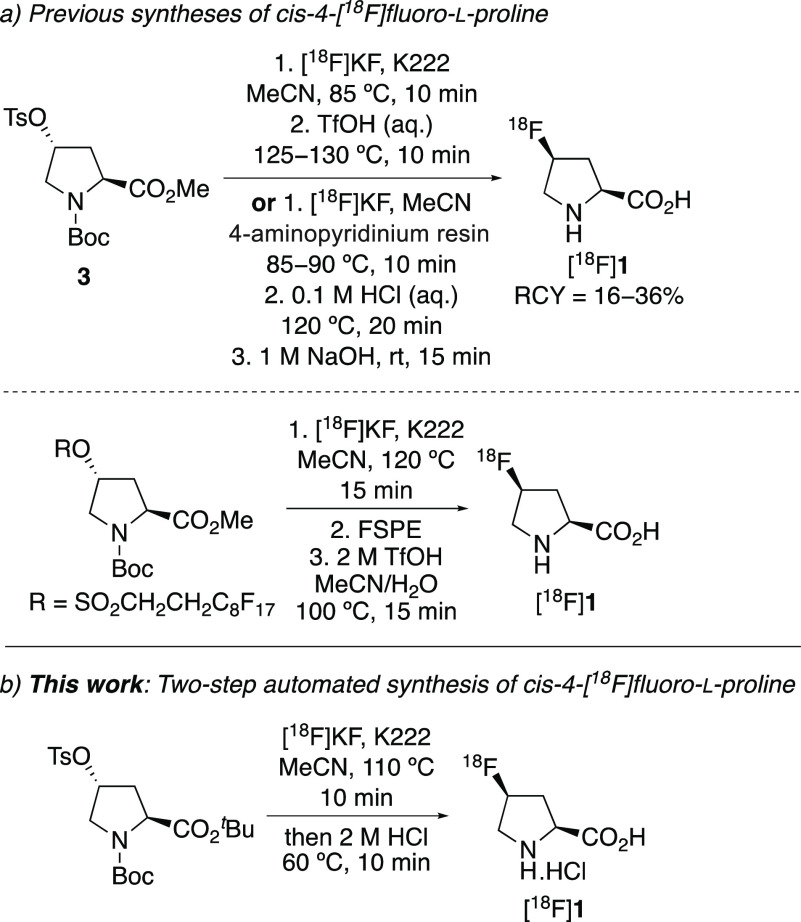
Synthesis of *cis*-4-[^18^F]Fluoro-l-proline

Due to the importance of *cis*- and *trans*-4-[^18^F]fluoro-l-proline ([^18^F]**1** and [^18^F]**2**, respectively), several methods for the radiosynthesis of these compounds have been developed.^4c^ The most common approach involves the reaction of 4-sulfonyloxy-l-proline derivatives with [^18^F]fluoride, leading to fluorination with inversion of configuration (Scheme 1a). Development of the fluorination step by automation has resulted in fast and efficient reactions, while formation of the undesired diastereomer (usually as a minor product) can be controlled by reaction temperature and removed by HPLC at the end of the process.^8^ The limitations of these approaches occur during the deprotection stage, which due to harsh conditions is performed manually. For example, removal of the Boc-protecting group and hydrolysis of the ester were done as a single step but required the use of triflic acid at 100–130 °C.^7a−7c^ Deprotection has also been done using a two-step strategy involving acid-mediated removal of the Boc group (0.1 M HCl, 120 °C), followed by hydrolysis of the methyl ester using sodium hydroxide.^7b^ In addition to requiring an extra step, alkaline hydrolysis of proline esters is known to produce side products, resulting in a decrease in the radiochemical yield (RCY).^7a^

For one of our imaging programs, we required access to *cis*- and *trans*-4-[^18^F]fluoro-l-proline ([^18^F]**1** and [^18^F]**2**, respectively) as well as the nonradioactive analogues as standards for radiochemistry studies. Due to the limitations of previous approaches, we sought to develop a fully automated synthesis involving both a nucleophilic radiofluorination reaction and a single-step deprotection process. We now report the nonradioactive synthesis of both *cis*- and *trans*-4-fluoro-l-proline (**1** and **2**, respectively) from readily available (2*S*,4*R*)-*N*-Boc-4-hydroxy-l-proline, using a deoxyfluorination reaction with morpholinosulfur trifluoride as the key step. Also described is a fully automated synthesis of [^18^F]**1** and [^18^F]**2**, which combines a highly effective nucleophilic radiofluorination with a single-step deprotection (Scheme 1b).

## Results and Discussion

Our primary aim during this project was the design and synthesis of proline derivatives that would undergo clean and efficient nucleophilic fluorination reactions and that could be deprotected in a single step, under mild conditions. Previous syntheses of 4-fluoroprolines have generally used an N-protected derivative of 4-hydroxyproline methyl ester as the starting material.^9,10^ However, issues were reported during the nucleophilic fluorination step involving intramolecular participation of the ester carbonyl, which led to the formation of a fluoroproline byproduct (17%) with retention of configuration.^11^ In this project, it was proposed that the use of a more bulky proline derivative, such as *N*-Boc-l-proline *tert*-butyl ester **5**, would minimize any intramolecular reactions during the fluorination step. Furthermore, the use of two acid-sensitive protecting groups would allow rapid and mild deprotection during the preparation of the ^18^F-labeled targets.

**Scheme 2:**
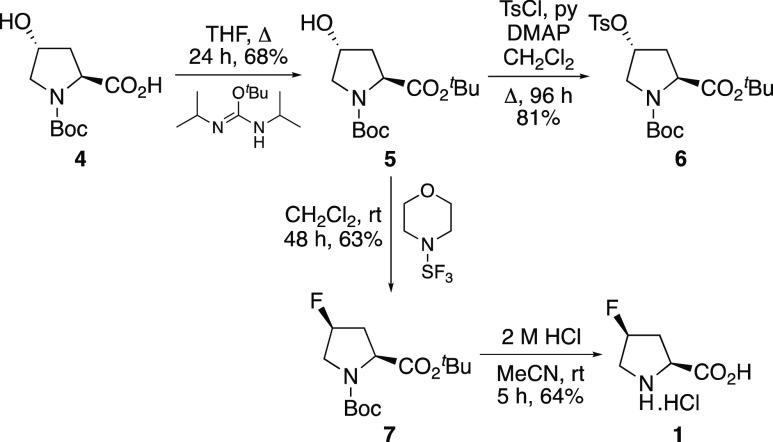
Synthesis of Precursor **6** and *cis*-4-Fluoro-l-proline (**1**)

Our synthesis of *cis*-4-fluoro-l-proline (**1**) began with the esterification of commercially available (2*S*,4*R*)-*N*-Boc-4-hydroxy-l-proline (**4**) with *O*-*tert*-butyl-*N*,*N*-diisopropylisourea (Scheme 2).^12^ This gave *tert*-butyl ester **5** in 68% yield. A precursor for radiofluorination studies and the synthesis of *cis*-4-[^18^F]fluoro-l-proline [^18^F]**1** was prepared by tosylation of **5** under standard conditions. An initial attempt to complete the synthesis of *cis*-4-fluoro-l-proline (**1**) investigated the nucleophilic fluorination of tosyl derivative **6** using TBAF.^10d^ However, this led to elimination of tosic acid and the isolation of dehydroproline derivatives. Instead, (2*S*,4*R*)-hydroxy-l-proline derivative **5** was treated with morpholinosulfur trifluoride, and this allowed the single-step synthesis of **7** in 63% yield. Analysis of the ^1^H NMR spectrum of the crude reaction material showed the presence of only the *cis* diastereomer, confirming complete inversion of configuration. This result suggests that the sterically encumbered *tert*-butyl ester prevents any intramolecular participation of the carbonyl and the formation of the undesired fluorinated *trans* diastereomer.^13^ Acid-mediated deprotection of **7** using 2 M hydrochloric acid at room temperature gave after recrystallization *cis*-4-fluoro-l-proline (**1**) in 64% yield.

**Scheme 3:**
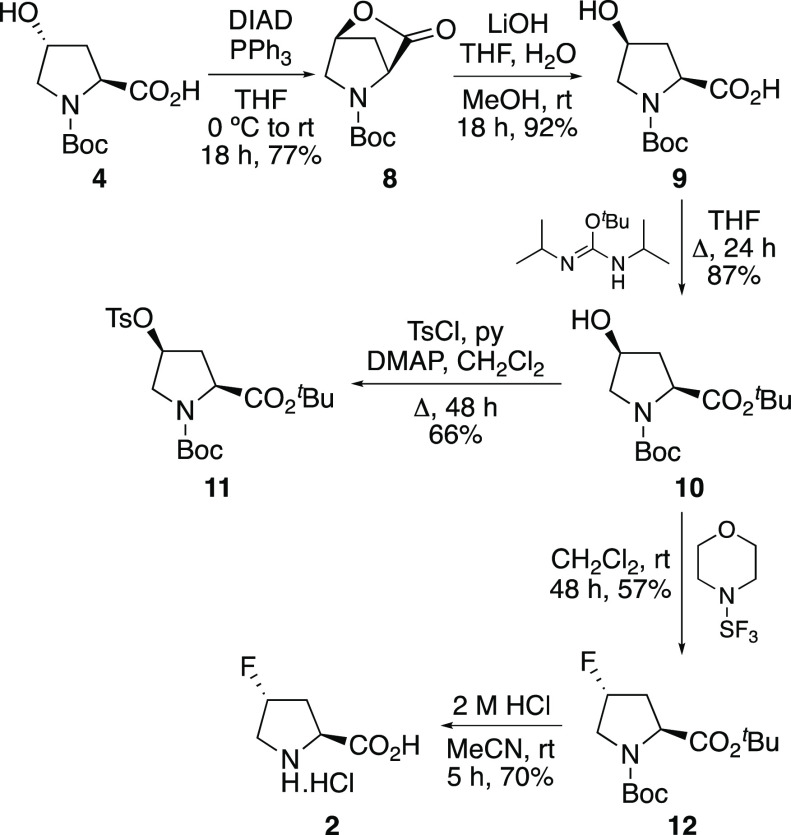
Synthesis of Precursor **11** and *trans*-4-Fluoro-l-proline (**2**)

To access *trans*-4-fluoro-l-proline (**2**) using the same approach required the preparation of (2*S*,4*S*)-*N*-Boc-4-hydroxy-l-proline (**9**). As (2*S*,4*R*)-*N*-Boc-4-hydroxy-l-proline (**4**) is readily available and inexpensive, we investigated a strategy for inversion of configuration of the 4-hydroxyl group. Previous methods have activated the 4*R*-hydroxyl group of (2*S*,4*R*)-4-hydroxy-l-proline ester derivatives by mesylation or using a Mitsunobu reaction, followed by inversion with benzoic acid and then hydrolysis of the resulting ester.^10c−10f^ Raines and co-workers described a three-step approach involving hydroxyl group mesylation, inversion by intramolecular lactonization with the α-carboxylic acid, and then lactone hydrolysis.^10d^ Inspired by this, we developed a two-step approach in which lactone **8** was initially prepared by an intramolecular Mitsunobu reaction of (2*S*,4*R*)-*N*-Boc-4-hydroxy-l-proline (**4**) (Scheme 3).^14^ Lactone **8** was then hydrolyzed at room temperature with lithium hydroxide to give (2*S*,4*S*)-*N*-Boc-4-hydroxy-l-proline (**9**) in 71% yield over the two steps. This approach was scalable, allowing the multigram synthesis of **9**. With the (2*S*,4*S*)-diastereomer **9** in hand, the same series of steps (*tert*-butyl esterification and tosylation) was used to access precursor **11**. Similarly, reaction of **10** with morpholinosulfur trifluoride gave 4-fluoroproline **12** as a single diastereomer, and deprotection under mild acidic conditions gave *trans*-4-fluoro-l-proline (**2**) in good overall yield.

**Scheme 4:**
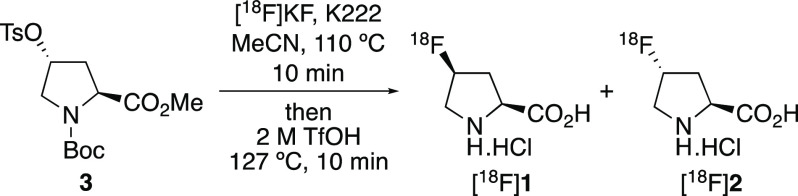
Automated Radiosynthesis of *cis*-4-[^18^F]Fluoro-l-proline [^18^F]**1** using Precursor **3**

The radiosynthesis of [^18^F]**1** and [^18^F]**2** using a TRACERlab FX_FN_ automated synthesizer and precursors **6** and **11** was next investigated. During these experiments, no-carrier-added [^18^F]fluoride from the cyclotron was trapped on a carbonate-preconditioned quaternary methylammonium (QMA) cartridge, eluted into the reactor with a solution containing K222/K_2_CO_3_, and then azeotropically dried. To compare the effectiveness of precursors **6** and **11** with previous methods, [^18^F]fluoride was reacted initially with commercially available (2*S*,4*R*)-proline methyl ester derivative **3** under literature conditions (Scheme 4). This involved reaction with [^18^F]fluoride at 110 °C for 10 min, followed by deprotection with 2 M triflic acid at 127 °C for 10 min.^7a,7c,8^ Although radio-HPLC analysis showed high conversion to *cis* isomer [^18^F]**1** (84.56%), *trans* isomer [^18^F]**2** (4.59%) and unreacted [^18^F]fluoride (10.85%) were also detected.^15^ In addition, multiple runs on the synthesizer using triflic acid caused damage to tubing and values, resulting in leaks and failed syntheses.

**Figure 2:**
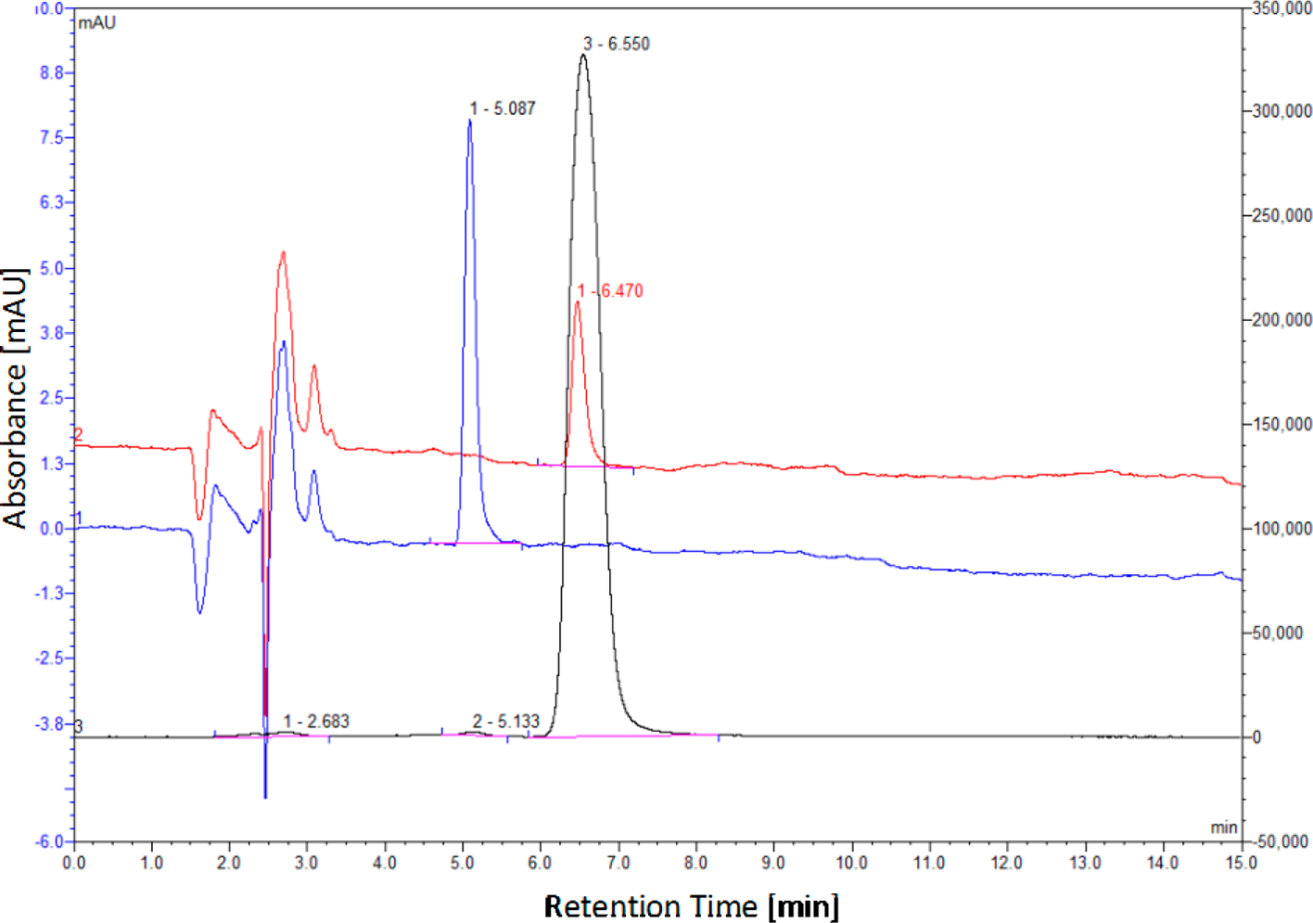
Radio-HPLC chromatogram of the reaction mixture (black). An overlay of the UV/vis HPLC trace of *trans*-4-fluoro-l-proline (**2**) (blue) and *cis*-4-fluoro-l-proline (**1**) (red) is also shown.

Similar conditions for radiofluorination and subsequent deprotection of (2*S*,4*R*)-proline *tert*-butyl ester derivative **6** were then investigated (Table 1, entry 1). To ensure complete conversion of [^18^F]fluoride, a longer radiofluorination reaction time of 15 min was used. In addition, triflic acid was replaced with 2 M hydrochloric acid during the deprotection stage. Following a total reaction time of 74 min, this gave [^18^F]**1** in a decay-corrected RCY of 42%. A benefit of a slightly longer radiofluorination reaction time was that less precursor was required for complete conversion of [^18^F]fluoride. With precursor **6**, the amount for each run could be reduced from 16 to 5 mg. The study next investigated the use of milder conditions to remove the acid-labile protecting groups. Radiofluorination of **6**, followed by deprotection with 2 M hydrochloric acid at 60 °C, gave [^18^F]**1** in 19% RCY (entry 2). It was proposed that the lower RCY for this production was partly due to the use of a strong cation exchange (SCX) cartridge during the final formulation stage. Therefore, the two-step process was repeated using both a shorter reaction time (5 min) for the deprotection step and a mixed-mode cation exchange (MCX) cartridge during the formulation (entry 3). This gave a 42% RCY of [^18^F]**1** after a total reaction time of 71 min. Further optimization was achieved by avoiding an evaporation stage after initial radiofluorination (entry 4). This resulted in a shorter overall reaction time of 63 min and gave [^18^F]**1** in 36% RCY. The corresponding radio-HPLC chromatogram under these optimized conditions showed clean synthesis of [^18^F]**1** (Figure 2). The reaction mixture was found to contain 98.8% [^18^F]**1**, with <0.4% *trans* isomer [^18^F]**2**.^15,16^

**Table 1:**
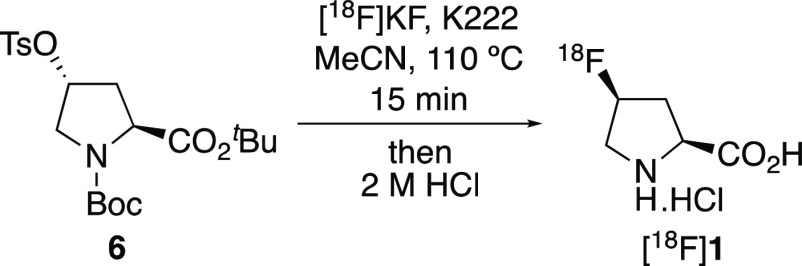
Optimization of the Automated Radiosynthesis of *cis*-4-[^18^F]Fluoro-l-proline [^18^F]**1**

entry	deprotection conditions	formulation cartridge	total reaction time (min)	RCY (%)^a^
1	127 °C, 10 min	SCX	74	42
2	60 °C, 10 min	SCX	66	19
3	60 °C, 5 min	MCX	71	42
4^b^	60 °C, 5 min	MCX	63	36

a Decay-corrected RCYs are presented.

b Evaporation was not performed after the fluorination step.

**Scheme 5:**
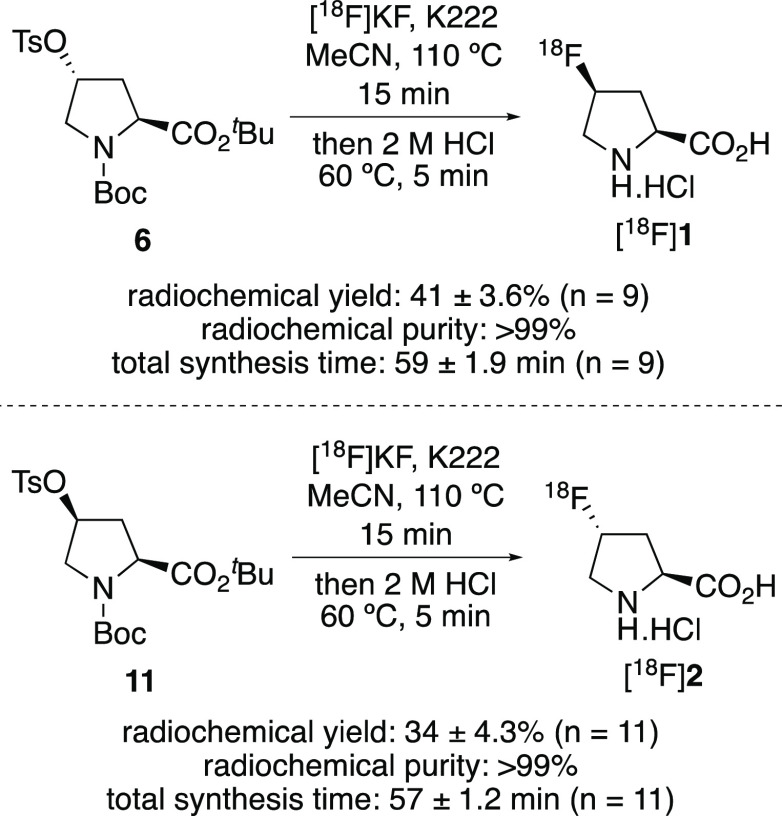
Automated Radiosynthesis of *cis*- and *trans*-4-[^18^F]Fluoro-l-proline ([^18^F]**1** and [^18^F]**2**, respectively) Decay-corrected RCYs are presented.

The optimized conditions were then used for the automated production, isolation, and purification of [^18^F]**1** (Scheme 5). After a total synthesis time of 59 min, this gave [^18^F]**1** in 41 ± 3.6% RCY (*n* = 9) with a >99% radiochemical purity. The molar activity of [^18^F]**1** was found to be >0.641 GBq μmol^–1^.^17^ The optimized conditions were then used for the automated synthesis of [^18^F]**2** using precursor **11**. In a similar manner, the two-step process was found to be highly selective for the preparation of [^18^F]**2**, generating the *trans* isomer in 97.7% yield, with 2.2% of the *cis* isomer also observed.^15^ Use of this method for the automated production and purification of [^18^F]**2** gave the target after a total synthesis time of 57 min, in 34 ± 4.3% RCY (*n* = 11) with a >99% radiochemical purity. The molar activity of [^18^F]**2** was found to be >0.320 GBq μmol^–1^.^17^ The stability of formulated products [^18^F]**1** and [^18^F]**2** using radio-HPLC was analyzed at 2 and 11 h points from the end of synthesis.^15^ For both isomers, there was no observed radiochemical byproduct after 11 h, which confirmed that these imaging agents are stable within this time frame to decomposition pathways, such as epimerization or radiolysis.

## Conclusions

In summary, a new approach for the preparation of *cis*-4-fluoro-l-proline (**1**) has been developed from (2*S*,4*R*)-*N*-Boc-4-hydroxy-l-proline (**4**). The use of a sterically hindered *tert*-butyl derivative during the key fluorination step with morpholinosulfur trifluoride prevented any intramolecular side reactions, yielding a single diastereomer as the sole product. Following preparation of (2*S*,4*S*)-*N*-Boc-4-hydroxy-l-proline (**9**) from commercially available (2*S*,4*R*)-diastereomer **4** via an intramolecular Mitsunobu reaction and lactone hydrolysis, a similar approach was developed for the preparation of *trans*-4-fluoro-l-proline (**2**). The tosylated derivatives were then investigated as substrates for a fully automated synthesis of *cis*- and *trans*-4-[^18^F]fluoro-l-proline ([^18^F]**1** and [^18^F]**2**, respectively). The bulky precursors underwent clean and reproducible radiofluorination, and the use of two acid-sensitive protecting groups allowed deprotection under mild conditions. It should be noted that both steps are highly amenable to automation when using a synthesizer and, thus, avoid typically harsh conditions and manual handling of radioactive intermediates.

## Experimental Section

All reagents and starting materials were obtained from commercial sources and used as received unless otherwise stated. Dry solvents were purified using a solvent purification system. Brine refers to a saturated solution of sodium chloride. All reactions were performed in oven-dried glassware under an atmosphere of argon unless otherwise stated. All mixtures for reactions performed at increased temperatures were heated using an oil bath. Flash column chromatography was carried out using silica gel (40–63 μm). Aluminum-backed plates precoated with silica gel 60 (UV_254_) were used for thin layer chromatography and visualized under ultraviolet light and by staining with KMnO_4_, ninhydrin, or vanillin. ^1^H NMR spectra were recorded on an NMR spectrometer at 400 or 500 MHz, and data are reported as follows: chemical shift in parts per million relative to tetramethylsilane or the solvent as the internal standard (CDCl_3_, δ 7.26), multiplicity (s, singlet; d, doublet; t, triplet; q, quartet; m, multiplet or overlap of nonequivalent resonances, integration). ^13^C{^1^H} NMR spectra were recorded on an NMR spectrometer at 101 or 126 MHz, and data are reported as follows: chemical shift in parts per million relative to tetramethylsilane or the solvent as the internal standard (CDCl_3_, δ 77.0), multiplicity with respect to hydrogen (deduced from DEPT experiments, C, CH, CH_2_, or CH_3_). IR spectra were recorded on a FTIR spectrometer; wavenumbers are indicated in inverse centimeters. Mass spectra were recorded using electron impact or electrospray ionization techniques. HRMS spectra were recorded using a dual-focusing magnetic analyzer mass spectrometer. Melting points are uncorrected. Optical rotations were determined as solutions irradiating with the sodium D line (λ = 589 nm) using a polarimeter. [α]_D_ values are given in units of 10^–1^ deg cm^2^ g^–1^.

### Di-*tert*-butyl (2*S*,4*R*)-4-Hydroxypyrrolidine-1,2-dicarboxylate (**5**)^18^

To a solution of *N*-(*tert*-butoxycarbonyl)-(2*S*,4*R*)-4-hydroxypyrrolidine-2-carboxylic acid (**4**) (0.500 g, 2.16 mmol) in dry tetrahydrofuran (2.5 mL), under argon at 0 °C was added *tert*-butyl *N,N*′-diisopropylcarbamimidate (0.500 mL, 2.16 mmol). The reaction mixture was heated to 70 °C for 3 h, followed by further addition of *tert*-butyl *N,N*′-diisopropylcarbamimidate (0.500 mL, 2.16 mmol). The reaction mixture was heated for a further 18 h. The reaction mixture was filtered through Celite and then concentrated *in vacuo*. Purification by flash column chromatography eluting with 50% ethyl acetate in hexane gave di-*tert*-butyl (2*S*,4*R*)-4-hydroxypyrrolidine-1,2-dicarboxylate (**5**) as a white solid (0.420 g, 68%): [α]_D_^14^ −55.3 (*c* 0.2, CHCl_3_) [lit.^18^ [α]_D_^25^ −51.3 (*c* 1.3, CHCl_3_)]. Spectroscopic data matched the literature.^18^

### Di-*tert*-butyl (2*S*,4*R*)-4-(Tosyloxy)pyrrolidine-1,2-dicarboxylate (**6**)^19^

To a solution of di-*tert*-butyl (2*S*,4*R*)-4-hydroxypyrrolidine-1,2-dicarboxylate (**5**) (1.50 g, 5.22 mmol) in dichloromethane (30 mL) at 0 °C were added pyridine (0.840 mL, 10.4 mmol), 4-dimethylaminopyridine (0.0640 g, 0.520 mmol), and *p*-toluenesulfonyl chloride (1.99 g, 10.4 mmol). The reaction mixture was heated to 40 °C for 96 h and then concentrated *in vacuo*. Purification by flash column chromatography eluting with 20% ethyl acetate in hexane gave di-*tert*-butyl (2*S*,4*R*)-4-(tosyloxy)pyrrolidine-1,2-dicarboxylate (**6**) as a white solid (1.90 g, 81%): [α]_D_^17^ −27.0 (*c* 0.1, CHCl_3_). Spectroscopic data matched the literature.^19^

### Di-*tert*-butyl (2*S*,4*S*)-4-Fluoropyrrolidine-1,2-dicarboxylate (**7**)

To a solution of di-*tert*-butyl (2*S*,4*R*)-4-hydroxypyrrolidine-1,2-dicarboxylate (**5**) (0.150 g, 0.522 mmol) in dry dichloromethane (3 mL), under argon, was added dropwise morpholinosulfur trifluoride (0.330 mL, 2.61 mmol). The reaction mixture was stirred at room temperature for 48 h and concentrated *in vacuo*, and water (20 mL) was added to the resulting residue. The aqueous layer was extracted with ethyl acetate (3 × 10 mL), and the combined extracts were then washed with water (3 × 10 mL) and sodium bicarbonate (3 × 10 mL), dried over MgSO_4_, filtered, and concentrated *in vacuo*. Purification by flash column chromatography eluting with 30% ethyl acetate in hexane gave di-*tert*-butyl (2*S*,4*S*)-4-fluoropyrrolidine-1,2-dicarboxylate (**7**) as a colorless oil (0.092 g, 63%): IR (neat) 2976, 1736, 1701, 1395, 1366, 1151, 1117, 1070, 769 cm^–1^; [α]_D_^15^ −33.3 (*c* 0.2, CHCl_3_); NMR spectra showed a 2:1 mixture of rotamers. Only data for the major rotamer were recorded: ^1^H NMR (500 MHz, CDCl_3_) δ 5.18 (dt, *J* = 53.0, 4.2 Hz, 1H), 4.29 (d, *J* = 9.3 Hz, 1H), 3.79 (dt, *J* = 27.0, 13.0 Hz, 1H), 3.73–3.57 (m, 1H), 2.51–2.20 (m, 2H), 1.48 (s, 9H), 1.45 (s, 9H); ^13^C{^1^H} NMR (126 MHz, CDCl_3_) δ 170.8 (C), 153.8 (C), 91.2 (d, ^1^*J*_C–F_ = 177.3 Hz, CH), 81.4 (C), 80.1 (C), 58.4 (CH), 53.0 (d, ^2^*J*_C–F_ = 24.6 Hz, CH_2_), 37.7 (d, ^2^*J*_C–F_ = 22.0 Hz, CH_2_), 28.3 (3 × CH_3_), 27.9 (3 × CH_3_); MS (ESI) *m*/*z* 312 (M + Na^+^, 100); HRMS (ESI) *m*/*z* [M + Na]^+^ calcd for C_14_H_24_FNNaO_4_ 312.1582, found 312.1579.

### (2*S*,4*S*)-4-Fluoropyrrolidine-2-carboxylic Acid Hydrochloride (**1**)

To a solution of di-*tert*-butyl (2*S*,4*S*)-4-fluoropyrrolidine-1,2-dicarboxylate (**7**) (0.0500 g, 0.170 mmol) in acetonitrile (0.2 mL) was added 2 M hydrochloric acid (2 mL). The reaction mixture was stirred at room temperature for 5 h and then concentrated *in vacuo*. Purification by trituration with chloroform yielded (2*S*,4*S*)-4-fluoropyrrolidine-2-carboxylic acid hydrochloride (**1**) as a white solid (0.0180 g, 64%): mp 130–136 °C dec; IR (neat) 3358, 2947, 1742, 1717, 1603, 1499, 1263, 1246, 1179, 1026 cm^–1^; [α]_D_^15^ −13.9 (*c* 0.1, MeOH); ^1^H NMR (500 MHz, CD_3_OD) δ 5.44 (dt, *J* = 52.1, 3.6 Hz, 1H), 4.65–4.59 (m, 1H), 3.74 (ddd, *J* = 20.0, 13.5, 1.5 Hz, 1H), 3.56 (ddd, *J* = 35.7, 13.5, 3.6 Hz, 1H), 2.78–2.58 (m, 2H); ^13^C{^1^H} NMR (126 MHz, CD_3_OD) δ 169.5 (C), 91.5 (d, ^1^*J*_C–F_ = 177.2 Hz, CH), 58.2 (CH), 52.0 (d, ^2^*J*_C–F_ = 24.0 Hz, CH_2_), 35.4 (d, ^2^*J*_C–F_ = 22.0 Hz, CH_2_); MS (ESI) *m*/*z* 134 (M + H^+^, 100); HRMS (ESI) *m*/*z* [M + H]^+^ calcd for C_5_H_9_FNO_2_ 134.0612, found 134.0611.

### *tert*-Butyl (1*S*,4*S*)-2-Oxa-3-oxo-5-azabicyclo[2.2.1]heptane-5-carboxylate (**8**)^20^

To a solution of *N*-(*tert*-butoxycarbonyl)-(2*S*,4*R*)-4-hydroxypyrrolidine-2-carboxylic acid (**4**) (6.00 g, 26.0 mmol) in dry tetrahydrofuran (200 mL) under argon at 0 °C was added triphenylphosphine (8.17 g, 31.1 mmol), followed by dropwise addition of diisopropyl azodicarboxylate (6.13 mL, 31.1 mmol). The reaction mixture was stirred at room temperature for 18 h and concentrated *in vacuo*. Purification by column chromatography eluting with 80% diethyl ether in hexane gave *tert*-butyl (1*S*,4*S*)-2-oxa-3-oxo-5-azabicyclo[2.2.1]heptane-5-carboxylate (**8**) as a white solid (4.30 g, 77%): [α]_D_^19^ +43.8 (*c* 1.0, CHCl_3_) [lit.^20^ [α]_D_^20^ +46.3 (*c* 1.0, CHCl_3_)]. Spectroscopic data matched the literature.^20^

### *N*-(*tert*-Butoxycarbonyl)-(2*S*,4*S*)-4-hydroxypyrrolidine-2-carboxylic Acid (**9**)^21^

To a solution of *tert*-butyl (1*S*,4*S*)-2-oxa-3-oxo-5-azabicyclo[2.2.1]heptane-5-carboxylate (**8**) (4.00 g, 18.8 mmol) in a mixture of water (60 mL), tetrahydrofuran (40 mL), and methanol (40 mL) was added lithium hydroxide monohydrate (2.36 g, 56.3 mmol). The reaction mixture was stirred at room temperature for 18 h and concentrated *in vacuo*, and ethyl acetate (100 mL) was added to the oily residue. The solution was acidified using a saturated aqueous solution of potassium hydrogen sulfate, and the aqueous layer extracted with ethyl acetate (3 × 150 mL). The combined extracts were dried over MgSO_4_ and concentrated *in vacuo* to give *N*-(*tert*-butoxycarbonyl)-(2*S*,4*S*)-4-hydroxypyrrolidine-2-carboxylic acid (**9**) as a white solid (4.00 g, 92%): [α]_D_^21^ −38.5 (*c* 0.3, MeOH) [lit.^21^ [α]_D_ −39.0 (*c* 0.7, MeOH)]. Spectroscopic data matched the literature.^21^

### Di-*tert*-butyl (2*S*,4*S*)-4-Hydroxypyrrolidine-1,2-dicarboxylate (**10**)^22^

Di-*tert*-butyl (2*S*,4*S*)-4-hydroxypyrrolidine-1,2-dicarboxylate (**10**) was prepared as described for di-*tert*-butyl (2*S*,4*R*)-4-hydroxypyrrolidine-1,2-dicarboxylate (**5**) using *N*-(*tert*-butoxycarbonyl)-(2*S*,4*S*)-4-hydroxypyrrolidine-2-carboxylic acid (**9**) (1.00 g, 4.32 mmol), dry tetrahydrofuran (5.0 mL), and *tert*-butyl *N,N*′-diisopropylcarbamimidate (0.965 mL, 4.33 mmol), followed by further addition of *tert*-butyl *N,N*′-diisopropylcarbamimidate (0.965 mL, 4.33 mmol) after 3 h. Purification by column chromatography eluting with 40% ethyl acetate in hexane gave di-*tert*-butyl (2*S*,4*S*)-4-hydroxypyrrolidine-1,2-dicarboxylate (**10**) as a white solid (0.650 g, 87%): [α]_D_^20^ −7.0 (*c* 0.1, CHCl_3_). Spectroscopic data matched the literature.^22^

### Di-*tert*-butyl (2*S*,4*S*)-4-(Tosyloxy)pyrrolidine-1,2-dicarboxylate (**11**)^22^

Di-*tert*-butyl (2*S*,4*S*)-4-(tosyloxy)pyrrolidine-1,2-dicarboxylate (**11**) was prepared as described for di-*tert*-butyl (2*S*,4*R*)-4-(tosyloxy)pyrrolidine-1,2-dicarboxylate (**6**) using di-*tert*-butyl (2*S*,4*S*)-4-hydroxypyrrolidine-1,2-dicarboxylate (**10**) (0.500 g, 1.74 mmol), dry dichloromethane (10 mL), pyridine (0.280 mL, 3.48 mmol), 4-dimethylaminopyridine (0.0210 g, 0.174 mmol), and *p*-toluenesulfonyl chloride (0.663 g, 3.48 mmol). The reaction mixture was heated for 48 h. Purification by column chromatography eluting with 20% ethyl acetate in hexane gave di-*tert*-butyl (2*S*,4*S*)-4-(tosyloxy)pyrrolidine-1,2-dicarboxylate (**11**) as a white solid (0.500 g, 66%): [α]_D_^20^ −25.4 (*c* 0.5, CHCl_3_) [lit.^22^ [α]_D_^34^ −28.3 (*c* 0.5, CHCl_3_)]. Spectroscopic data matched the literature.^22^

### Di-*tert*-butyl (2*S*,4*R*)-4-Fluoropyrrolidine-1,2-dicarboxylate (**12**)

Di-*tert*-butyl (2*S*,4*R*)-4-fluoropyrrolidine-1,2-dicarboxylate (**12**) was prepared as described for di-*tert*-butyl (2*S*,4*S*)-4-fluoropyrrolidine-1,2-dicarboxylate (**7**) using di-*tert*-butyl (2*S*,4*S*)-4-hydroxypyrrolidine-1,2-dicarboxylate (**10**) (0.150 g, 0.522 mmol), dry dichloromethane (3 mL), and morpholinosulfur trifluoride (0.330 mL, 2.61 mmol). Purification by column chromatography eluting with 20% ethyl acetate in hexane gave di-*tert*-butyl (2*S*,4*R*)-4-fluoropyrrolidine-1,2-dicarboxylate (**12**) as a colorless oil (0.084 g, 57%): IR (neat) 2978, 1744, 1703, 1398, 1368, 1152 cm^–1^; [α]_D_^20^ −8.8 (*c* 0.2, CHCl_3_). NMR spectra showed a 2:1 mixture of rotamers; only data for the major rotamer were recorded: ^1^H NMR (400 MHz, CDCl_3_) δ 5.16 (dt, *J* = 52.6, 3.0 Hz, 1H), 4.26 (t, *J* = 8.3 Hz, 1H), 3.89 (ddd, *J* = 21.8, 13.0, 3.0 Hz, 1H), 3.55 (ddd, *J* = 36.1, 13.0, 3.0 Hz, 1H), 2.64–2.44 (m, 1H), 2.14–1.92 (m, 1H), 1.44 (s, 9H), 1.42 (s, 9H); ^13^C{^1^H} NMR (101 MHz, CDCl_3_) δ 171.7 (C), 153.8 (C), 91.0 (d, ^1^*J*_C–F_ = 178.7 Hz, CH), 81.4 (C), 80.4 (C), 58.2 (CH), 53.0 (d, ^2^*J*_C–F_ = 22.8 Hz, CH_2_), 37.6 (d, ^2^*J*_C–F_ = 22.8 Hz, CH_2_), 28.3 (3 × CH_3_), 28.0 (3 × CH_3_); MS (ESI) *m*/*z* 312 (M + Na^+^, 100); HRMS (ESI) *m*/*z* [M + Na]^+^ calcd for C_14_H_24_FNNaO_4_ 312.1582, found 312.1580.

### (2*S*,4*R*)-4-Fluoropyrrolidine-2-carboxylic Acid Hydrochloride (**2**)

(2*S*,4*R*)-4-Fluoropyrrolidine-2-carboxylic acid hydrochloride (**2**) was prepared as described for (2*S*,4*S*)-4-fluoropyrrolidine-2-carboxylic acid hydrochloride (**1**) using di-*tert*-butyl (2*S*,4*R*)-4-fluoropyrrolidine-1,2-dicarboxylate (**12**) (0.0800 g, 0.280 mmol), acetonitrile (0.25 mL), and 2 M hydrochloric acid (2.5 mL). This gave (2*S*,4*S*)-4-fluoropyrrolidine-2-carboxylic acid hydrochloride (**2**) as an off-white solid (0.0331 g, 70%): mp 148–152 °C dec; IR (neat) 3672, 2987, 1738, 1682, 1406, 1242, 1220, 1067, 1051 cm^–1^; [α]_D_^17^ −6.5 (*c* 0.1, MeOH); ^1^H NMR (400 MHz, CD_3_OD) δ 5.47 (dt, *J* = 51.8, 3.6 Hz, 1H), 4.61 (dd, *J* = 10.5, 7.9 Hz, 1H), 3.73–3.52 (m, 2H), 2.84–2.70 (m, 1H), 2.39 (dddd, *J* = 38.5, 14.8, 10.5, 3.6 Hz, 1H); ^13^C{^1^H} NMR (101 MHz, CD_3_OD) δ 169.3 (C), 92.0 (d, ^1^*J*_C–F_ = 177.0 Hz, CH), 58.0 (CH), 51.7 (d, ^2^*J*_C–F_ = 24.0 Hz, CH_2_), 35.4 (d, ^2^*J*_C–F_ = 22.1 Hz, CH_2_); MS (ESI) *m*/*z* 134 (M + H^+^, 100); HRMS (ESI) *m*/*z* [M + H]^+^ calcd for C_5_H_9_FNO_2_ 134.0612, found 134.0613.

### Radiochemistry: General Experimental

No-carrier-added aqueous [^18^F]fluoride was produced via the ^18^O(p,n)^18^F nuclear reaction by irradiation of ^18^O-enriched water by a GE PETtrace 8 cyclotron. All radiofluorination reactions were carried out on a GE TRACERlab FX_FN_ automated synthesizer. Sep-Pak QMA Carbonate Plus Light cartridges (Waters) were preconditioned with water (10 mL) prior to use. Oasis MCX Plus Short (Waters) and Bond Elut SCX 1 g (Agilent) cartridges were preconditioned with ethanol (5 mL) and then with water (10 mL) prior to use. The starting activity for calculating the radiochemical yield was determined from the GM reading taken immediately following delivery of [^18^F]fluoride to the synthesizer from the cyclotron. The final activity readings were recorded using a Capintec CRC-25 PET dose calibrator.

### Analytical HPLC Method

Analytical HPLC was carried out on a Thermo Dionex Ulimate system 3000 equipped with a Berthold FlowStar LB 513 radio flow detector and a DAD-3000 UV detector. An isocratic mobile phase of 60% acetonitrile in water was used with a Phenomenex Luna 5 μm NH_2_ 100 Å, 250 mm × 4.6 mm column at a rate of 1 mL min^–1^. The nonradioactive standards were detected using a UV wavelength of 210 nm.

### *cis*-4-[^18^F]Fluoro-l-proline [^18^F]**1**

Cyclotron target water containing [^18^F]fluoride was transferred to and trapped on a Sep-Pak QMA Carbonate Plus Light cartridge. The activity was eluted into a reaction vessel using a solution of Kryptofix 222 (15 mg) and potassium carbonate (2.4 mg) in acetonitrile (0.80 mL) and water (0.40 mL). This solution was dried by being stirred at 100 °C under vacuum and a stream of helium gas for 2 min. This process was repeated twice using acetonitrile (2 × 1 mL). The [^18^F]fluoride was then completely dried by applying full vacuum for 1 min. Di-*tert*-butyl (2*S*,4*R*)-4-(tosyloxy)pyrrolidine-1,2-dicarboxylate (**6**) (5.0 mg) in acetonitrile (1.0 mL) was added to the reaction vessel, which was sealed, and the mixture heated to 110 °C for 15 min while being stirred. The reaction mixture was then cooled to 60 °C, and a 4 M aqueous solution of hydrochloric acid (1.0 mL) was added (resulting in a 2 M concentration of hydrochloric acid). The reaction mixture was stirred at this temperature for 5 min and then concentrated by applying vacuum under a stream of helium gas. The resultant residue was then cooled to 30 °C and diluted with a 50% aqueous solution of acetonitrile (2.0 mL). The reaction mixture was then transferred into the HPLC injector loop for purification. Purification was performed by semipreparative HPLC with a SYKMN S1122 solvent delivery system using a Phenomenex Luna 5 μm NH_2_ 100 Å, 250 mm × 10 mm column and eluted using a 60% aqueous solution of acetonitrile at a flow rate of 4 mL min^–1^. The product fraction was identified using a gamma detector at a retention time of approximately 9 min and collected into a flask containing an aqueous solution (20 mL) adjusted to pH 3 using phosphoric acid. The diluted fraction was then passed onto an Oasis MCX Plus Short cartridge, washed with water (10 mL), and eluted from the cartridge with a 0.1 M aqueous solution of sodium phosphate (6.0 mL). The formulation was then adjusted to pH 7 by the addition of a 1 M aqueous solution of hydrochloric acid (0.5 mL). *cis*-4-[^18^F]Fluoro-l-proline [^18^F]**1** was isolated in 41 ± 3.6% radiochemical yield with a radiochemical purity of >99% (*n* = 9). The total synthesis time from delivery of [^18^F]fluoride to extraction of the product was 59 ± 1.9 min.

### *trans*-4-[^18^F]Fluoro-l-proline [^18^F]**2**

The reaction was carried out according to the same general procedure as that for *cis*-4-[^18^F]fluoro-l-proline [^18^F]**1** using di-*tert*-butyl (2*S*,4*S*)-4-(tosyloxy)pyrrolidine-1,2-dicarboxylate (**11**) (5.0 mg) in acetonitrile (1.0 mL). The product fraction was identified using a gamma detector at a retention time of approximately 7 min. *trans*-4-[^18^F]Fluoro-l-proline [^18^F]**2** was isolated in 34 ± 4.3% radiochemical yield with a radiochemical purity of >99% (*n* = 11). The total synthesis time from delivery of [^18^F]fluoride to extraction of the product was 57 ± 1.2 min.

## Supporting Information Available

HPLC chromatograms and ^1^H and ^13^C{^1^H} NMR spectra of all compounds (PDF)
