# Supplementary material for: Automated Radiosynthesis of cis- and trans-4-[18F]Fluoro-l-proline Using [18F]Fluoride
Source: J Org Chem. 2021 Apr 29;86(20):14054–60. doi: 10.1021/acs.joc.1c00755 (PMC8524414; doi:10.1021/acs.joc.1c00755)
Supplement: Supplementary file 1 — jo1c00755_si_001.pdf [file jo1c00755_si_001.pdf]

Supporting Information for:

Automated Radiosynthesis of *cis*- and *trans*-4-[^{18}F]Fluoro-L-proline using [^{18}F]Fluoride

Timaeus E. F. Morgan,[‡] Leanne M. Riley,[†] Adriana A. S. Tavares[‡] and Andrew Sutherland*[†]

[‡]University/BHF Centre for Cardiovascular Science, University of Edinburgh, Edinburgh EH16 4TJ, United Kingdom. [†]WestCHEM, School of Chemistry, The Joseph Black Building, University of Glasgow, Glasgow G12 8QQ, United Kingdom.

Table of Contents

1. HPLC Chromatograms of All Compounds S2–S6
2. Stability Measurements Post Radiosynthesis of [^{18}F]**1** and [^{18}F]**2** S7
3. ^{1}H and ^{13}C{^{1}H} NMR Spectra of All Compounds S8–S27

1. HPLC Chromatograms of All Compounds

a) Synthesis of *cis*-4-[^{18}F]fluoro-L-proline [^{18}F]1 using precursor 3.

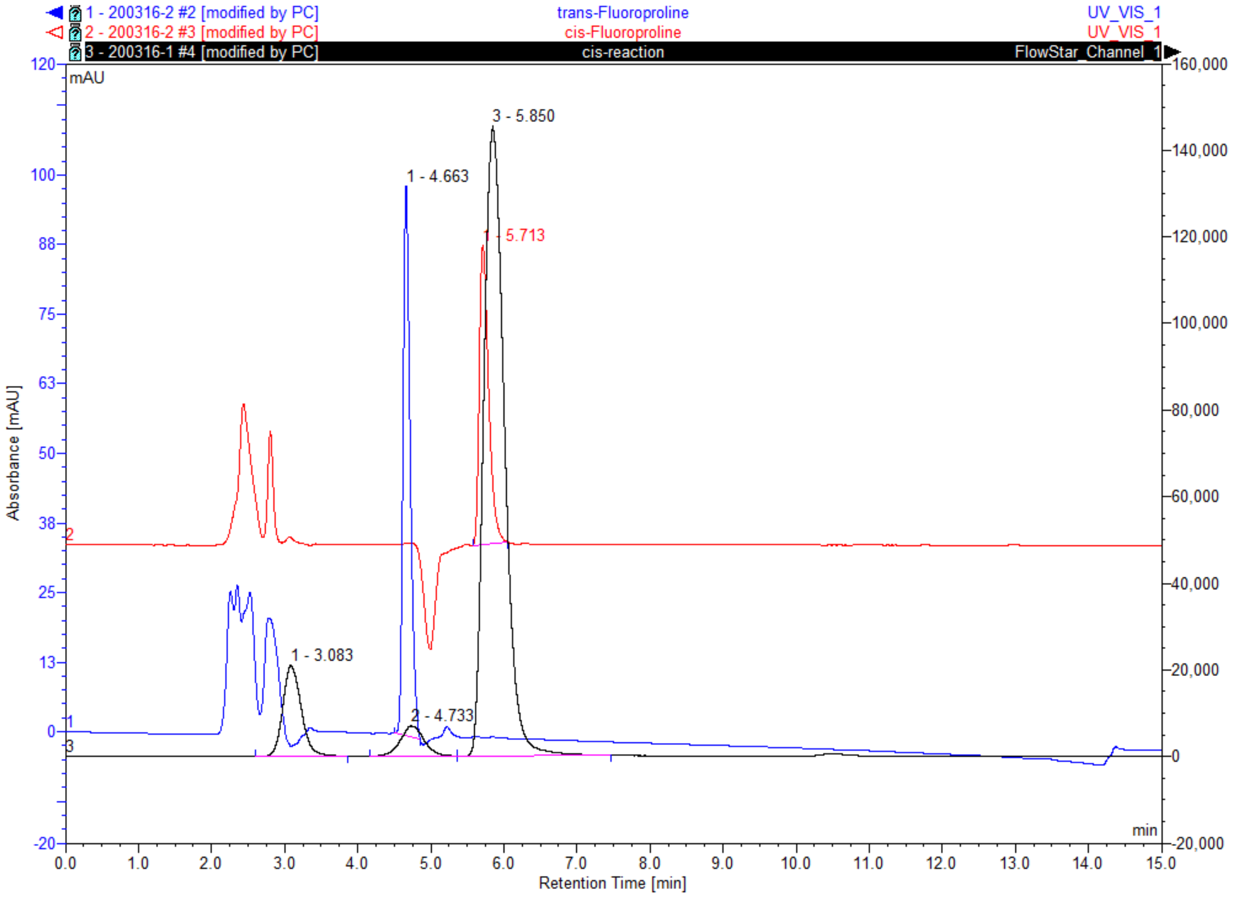

No.	Peakname	Ret.Time min	Area *min	Amount	Type	Height	Rel.Area %	Resolution
1	n.a.	3.083	#########	n.a.	BMB*	##########	10.85	3.23
2	n.a.	4.733	#########	n.a.	BMb*	6966.539	4.59	2.06
3	n.a.	5.850	#########	n.a.	bMB*	##########	84.56	n.a.
Total:			#########	0.0000		##########	100.00	

Figure S1: Radio-HPLC of reaction mixture (black); UV trace of *trans*-4-fluoro-L-proline (**2**) (blue); UV trace of *cis*-4-fluoro-L-proline (**1**) (red).

b) Synthesis of *cis*-4-[^{18}F]fluoro-L-proline [^{18}F]1 using precursor 6.

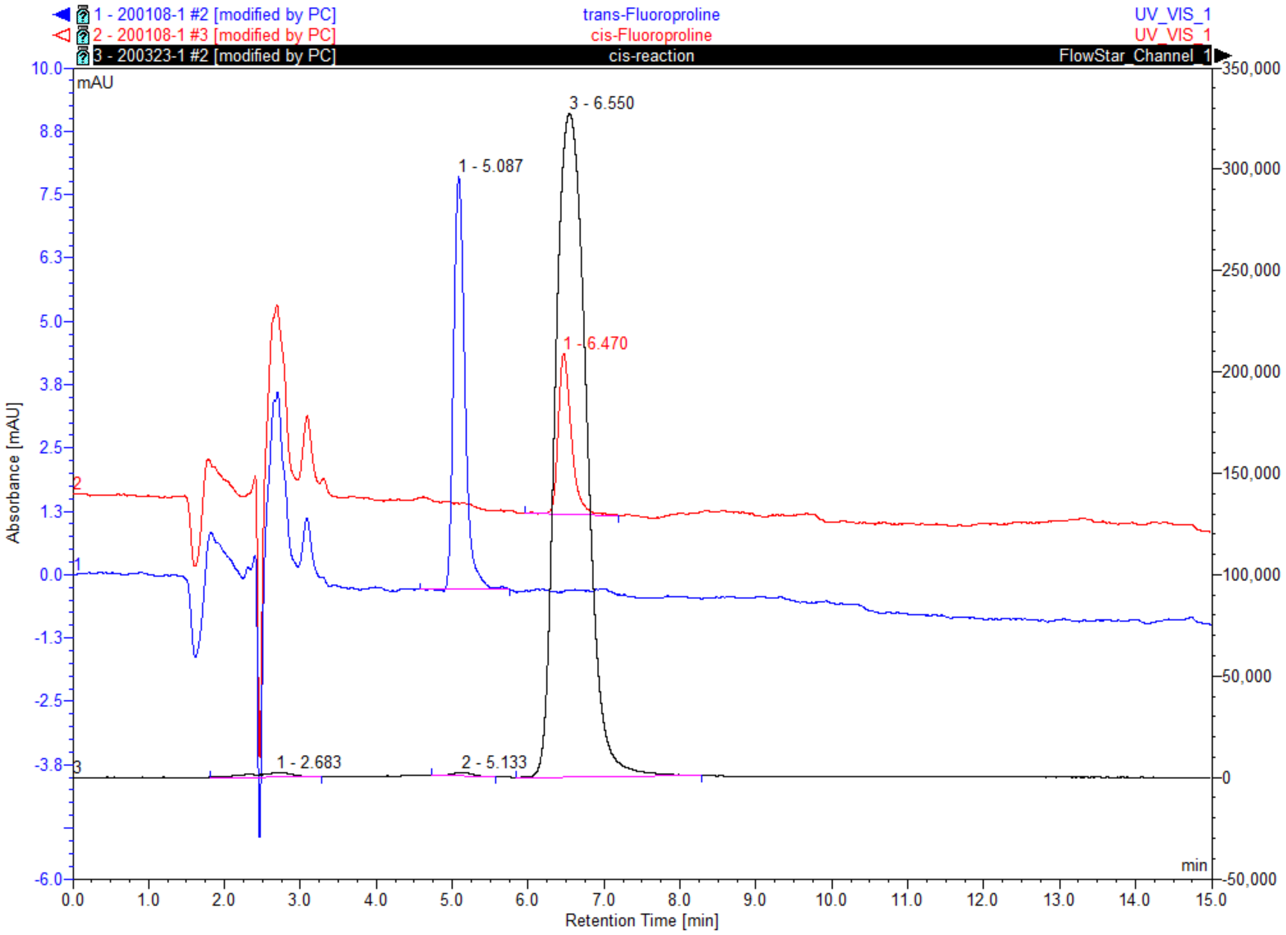

No.	Peakname	Ret.Time min	Area *min	Amount	Type	Height	Rel.Area %	Resolution
1	n.a.	2.683	##########	n.a.	BMB*	2150.435	0.83	2.91
2	n.a.	5.133	573.7252	n.a.	BMB*	1907.625	0.37	2.26
3	n.a.	6.550	##########	n.a.	BMB*	##########	98.80	n.a.
Total:			##########	0.0000		##########	100.00	

Figure S2: Radio-HPLC of reaction mixture (black); UV trace of *trans*-4-fluoro-L-proline (**2**) (blue); UV trace of *cis*-4-fluoro-L-proline (**1**) (red).

c) Synthesis of *trans*-4-[^{18}F]fluoro-L-proline [^{18}F]2 using precursor 11.

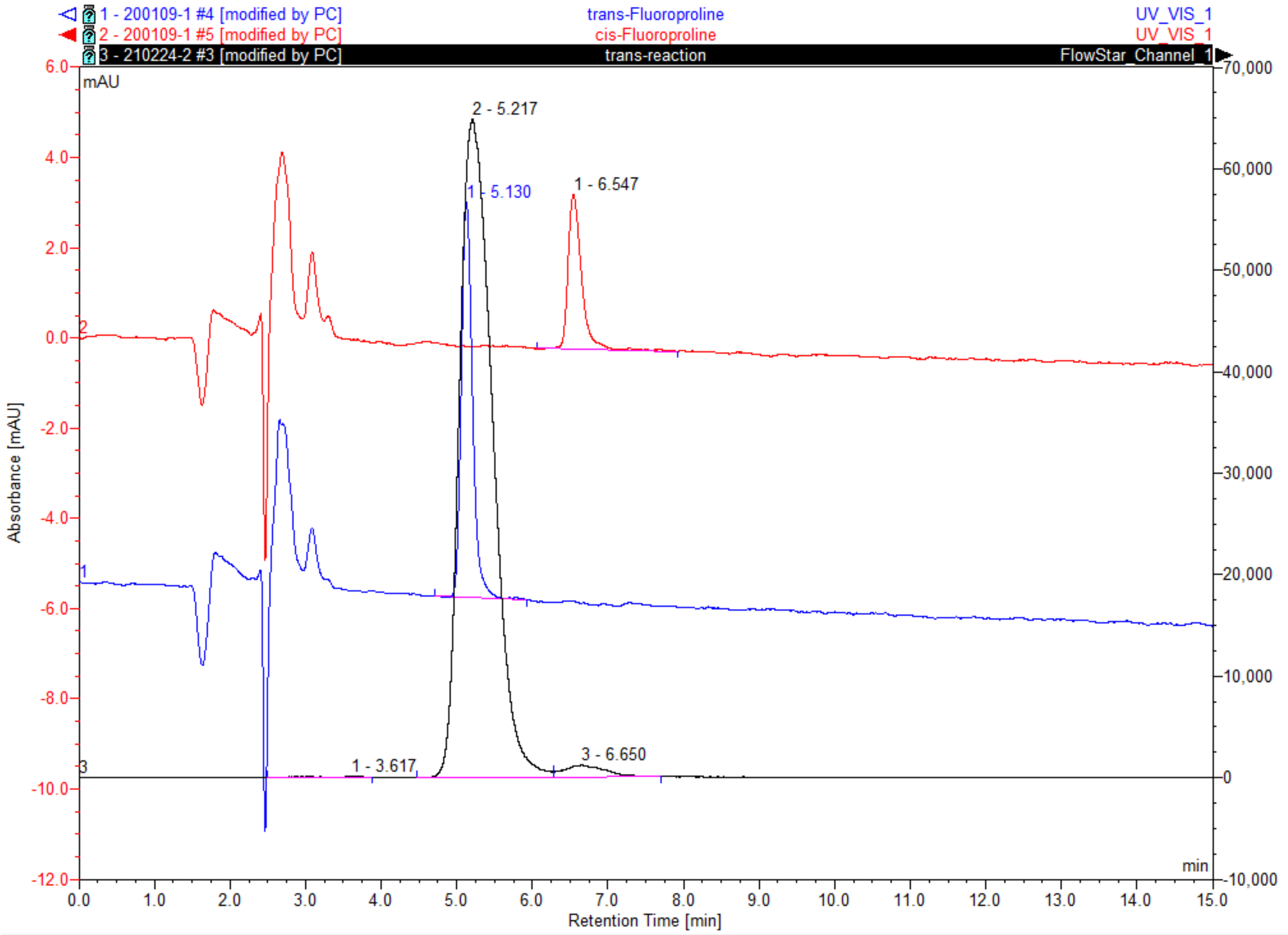

No.	Peakname	Ret.Time min	Area *min	Amount	Type	Height	Rel.Area %	Resolution
1	n.a.	3.617	49.6370	n.a.	BMB*	82.516	0.15	1.27
2	n.a.	5.217	#########	n.a.	BM *	##########	97.65	1.53
3	n.a.	6.650	739.5055	n.a.	MB*	1187.788	2.20	n.a.
Total:			#########	0.0000		##########	100.00	

Figure S3: Radio-HPLC of reaction mixture (black); UV trace of *trans*-4-fluoro-L-proline (**2**) (blue); UV trace of *cis*-4-fluoro-L-proline (**1**) (red).

d) Synthesis of *cis*-4-[^{18}F]fluoro-L-proline [^{18}F]1

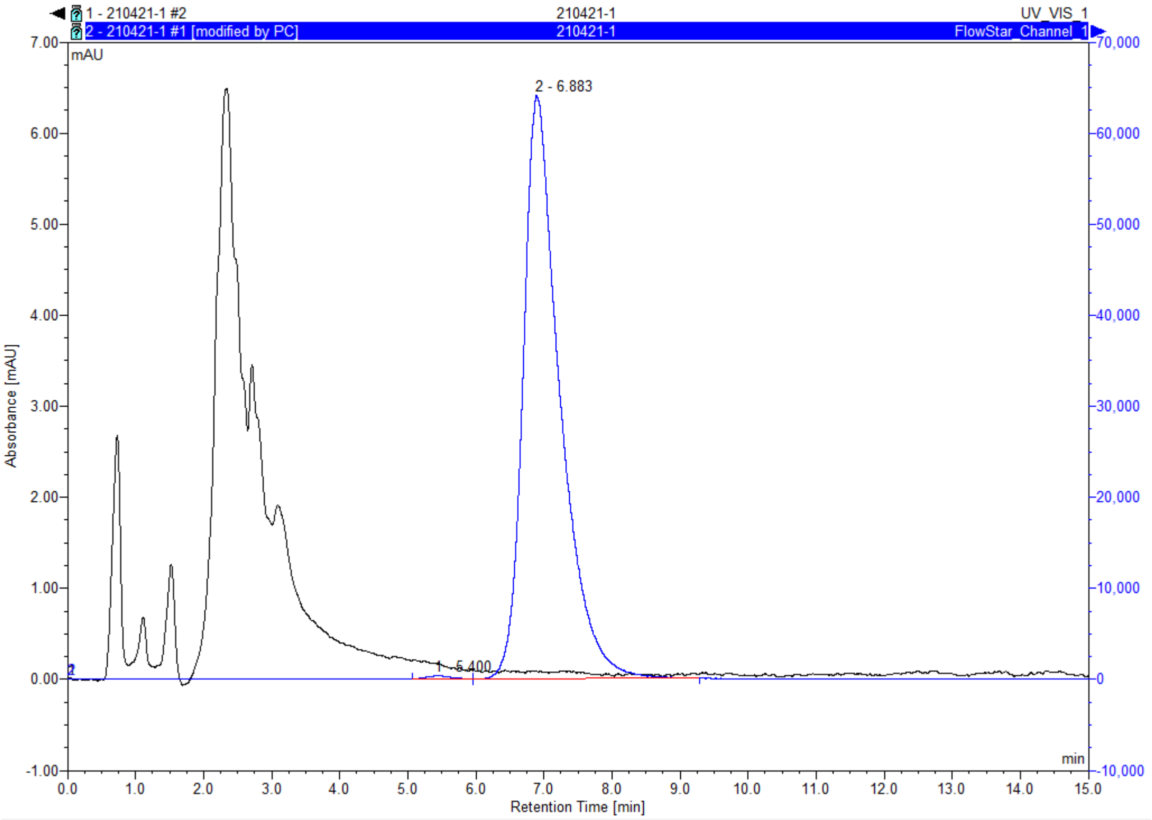

Figure S4: HPLC traces for determination of molar activity of [^{18}F]**1**. Radio-HPLC (blue); UV trace (black).

e) Synthesis of *trans*-4-[^{18}F]fluoro-L-proline [^{18}F]2

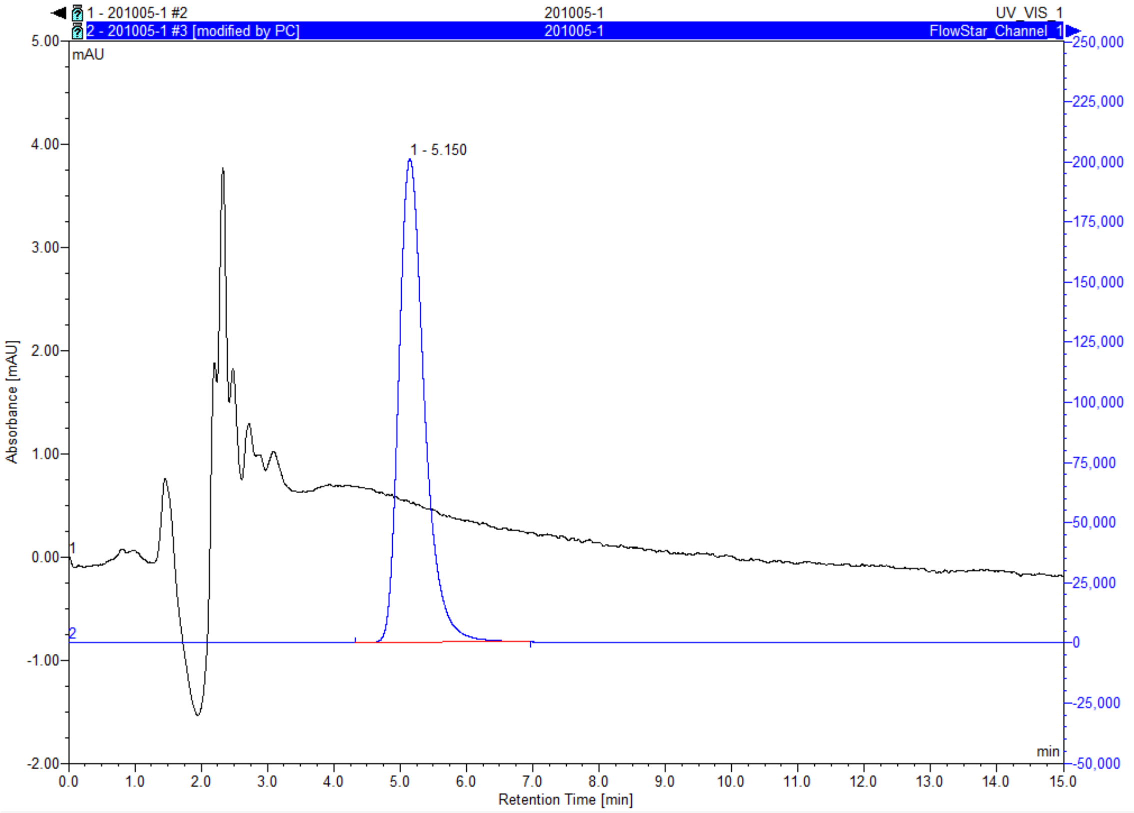

Figure S5: HPLC traces for determination of molar activity of [^{18}F]**2**. Radio-HPLC (blue); UV trace (black).

2. Stability Measurements Post Radiosynthesis of [^{18}F]1 and [^{18}F]2

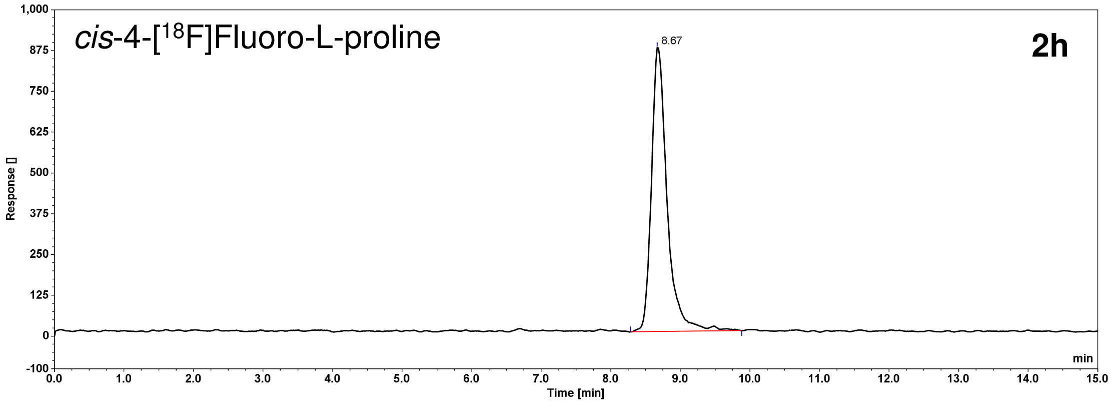

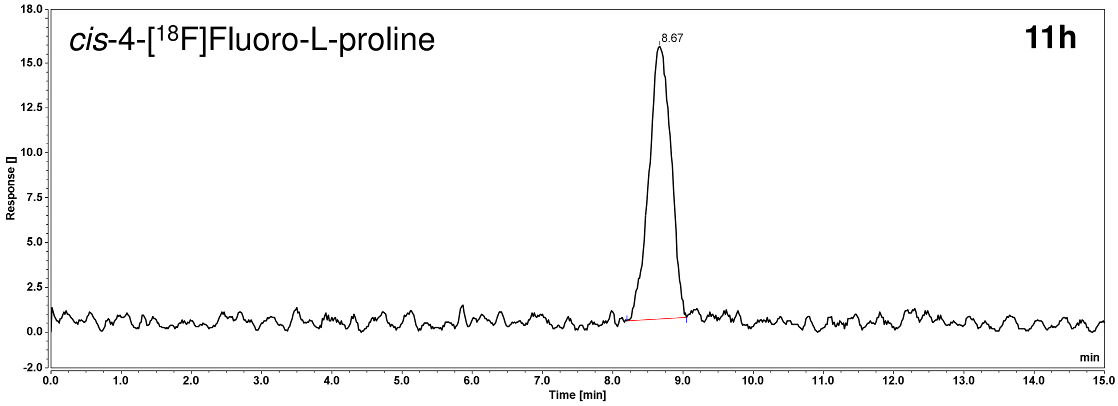

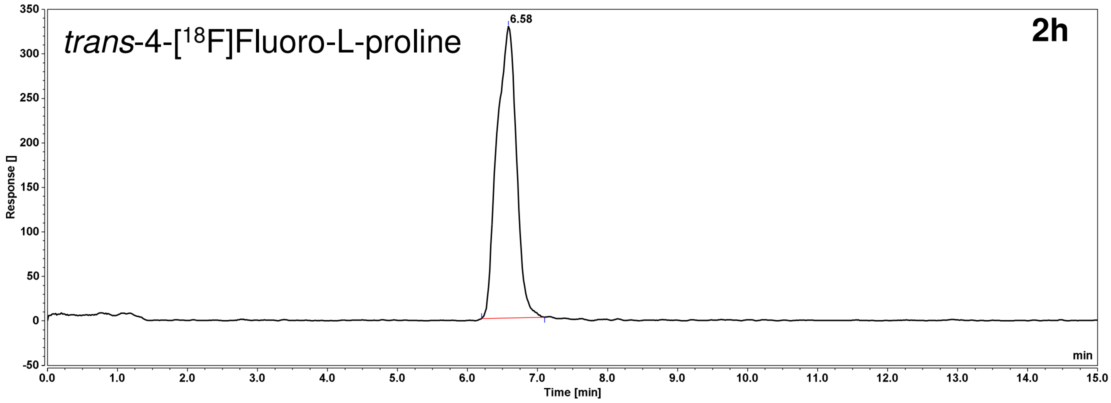

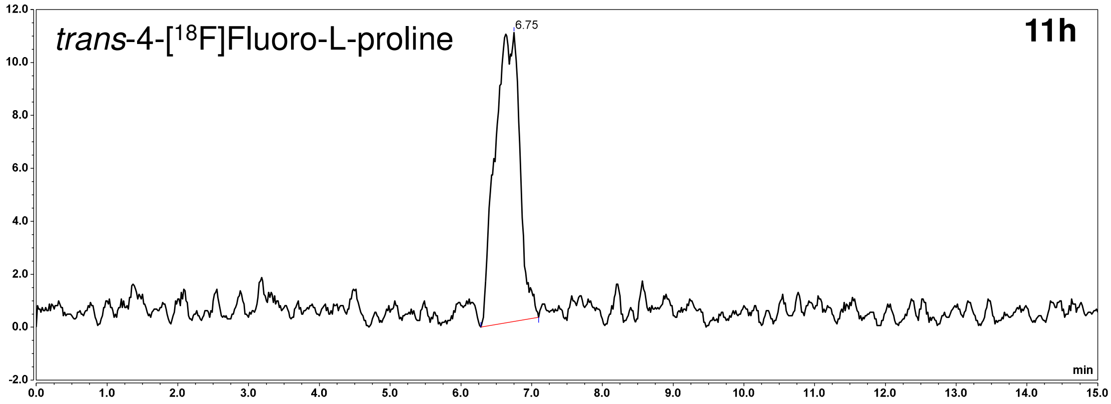

3. ¹H and $^{13}C\{^1H\}$ NMR Spectra of All Compounds

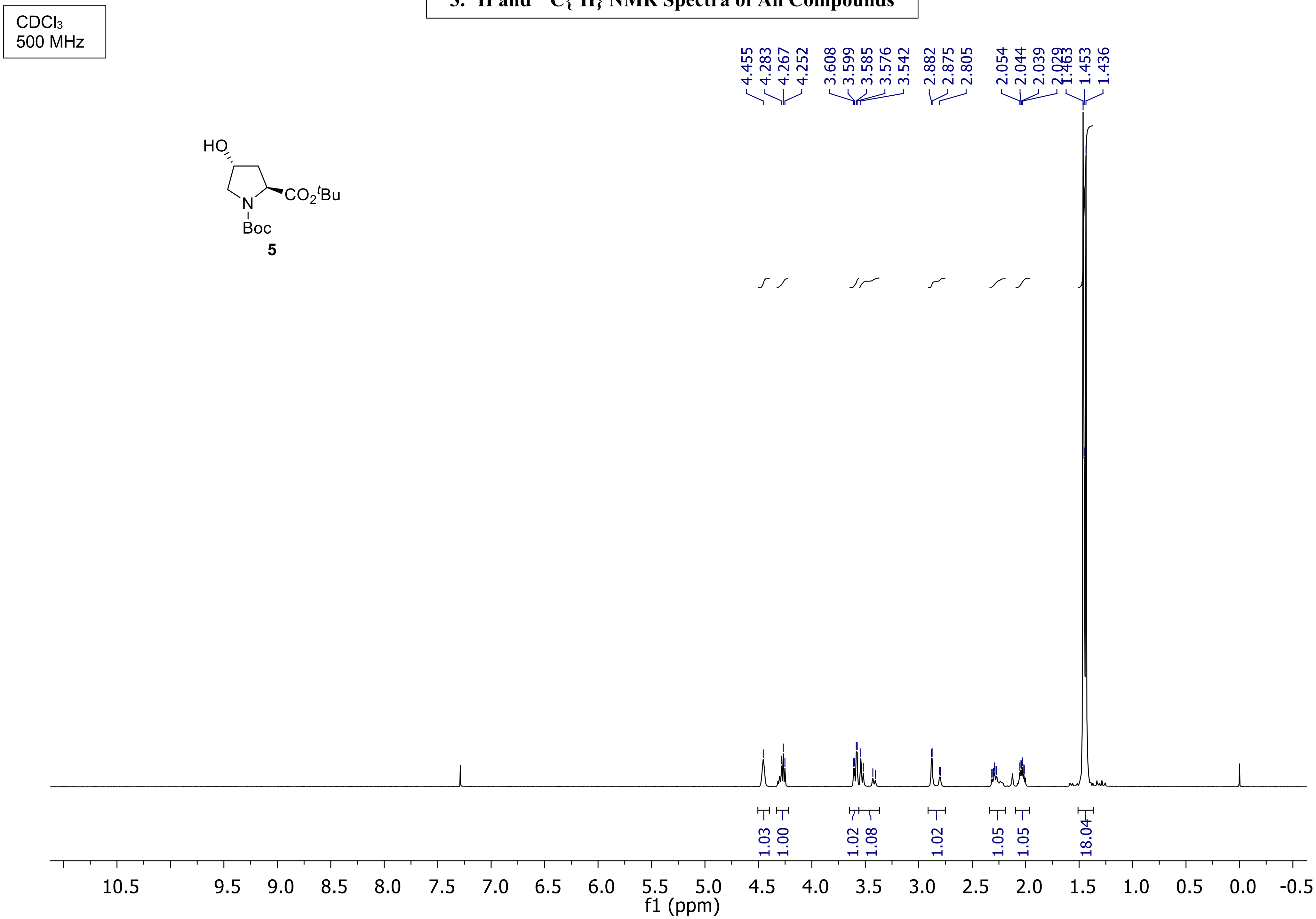

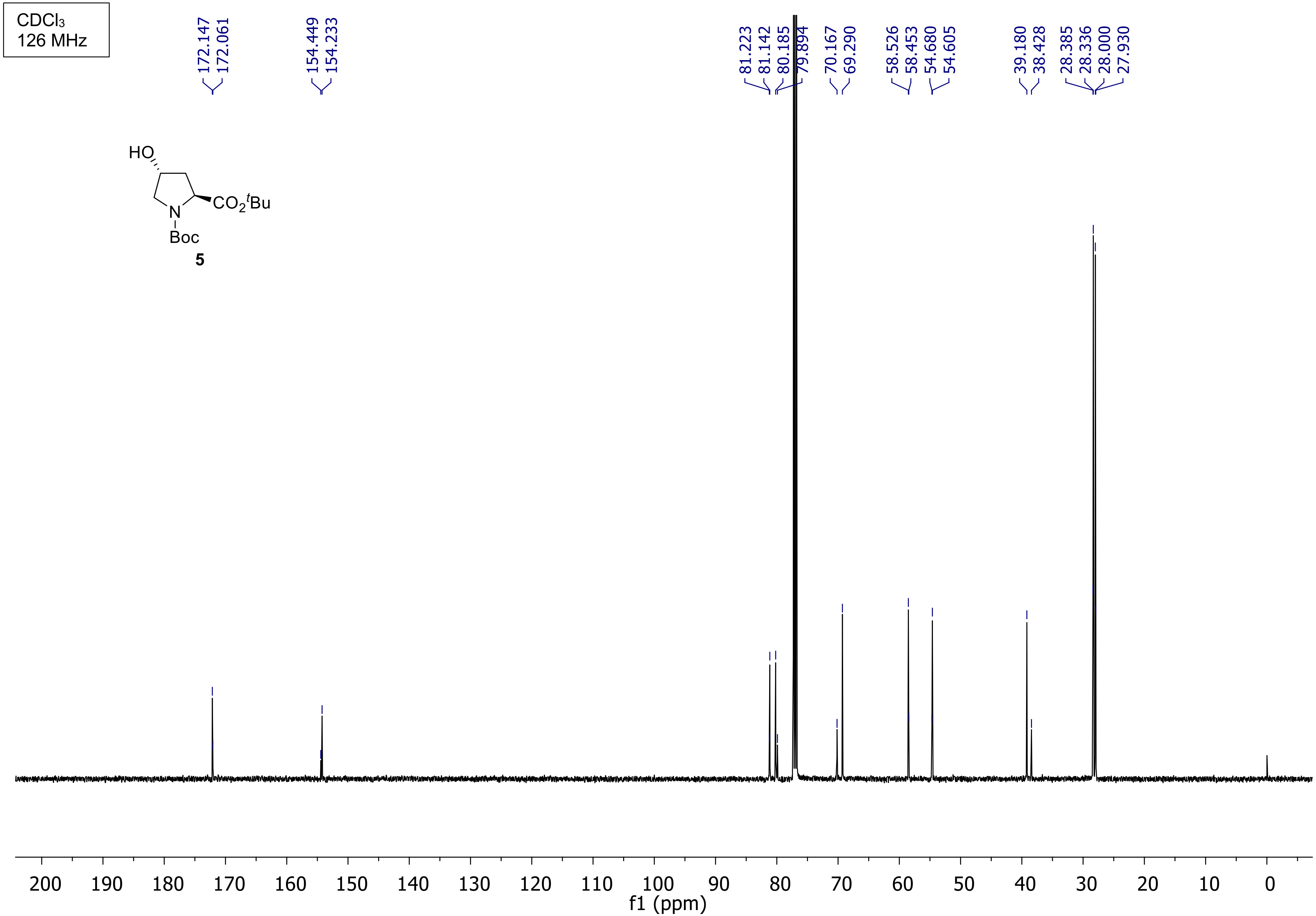
CDCl3
126 MHz
172.147
172.061
154.449
154.233
81.223
81.142
80.185
79.894
70.167
69.290
58.526
58.453
54.680
54.605
39.180
38.428
28.385
28.336
28.000
27.930
HO
CO2tBu
N
Boc
5
200 190 180 170 160 150 140 130 120 110 100 90 80 70 60 50 40 30 20 10 0
f1 (ppm)

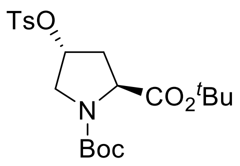

6

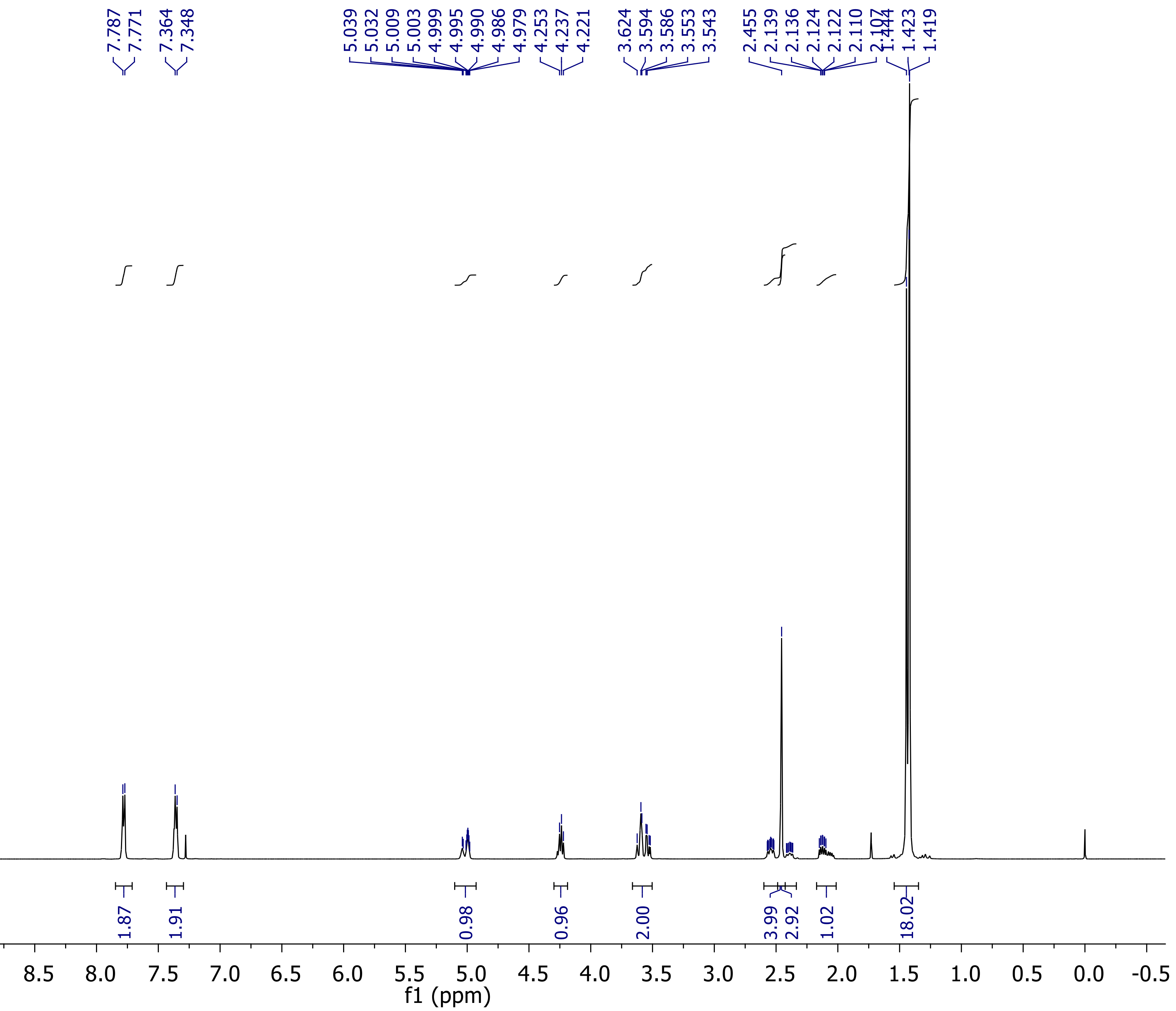

CDCl3
500 MHz
7.787
7.771
7.364
7.348
5.039
5.032
5.009
5.003
4.999
4.995
4.990
4.986
4.979
4.253
4.237
4.221
3.624
3.594
3.586
3.553
3.543
2.455
2.139
2.136
2.124
2.122
2.110
2.107
1.444
1.423
1.419
1.87
1.91
0.98
0.96
2.00
3.99
2.92
1.02
18.02
10.5
9.5
9.0
8.5
8.0
7.5
7.0
6.5
6.0
5.5
5.0
4.5
4.0
3.5
3.0
2.5
2.0
1.5
1.0
0.5
0.0
-0.5
f1 (ppm)

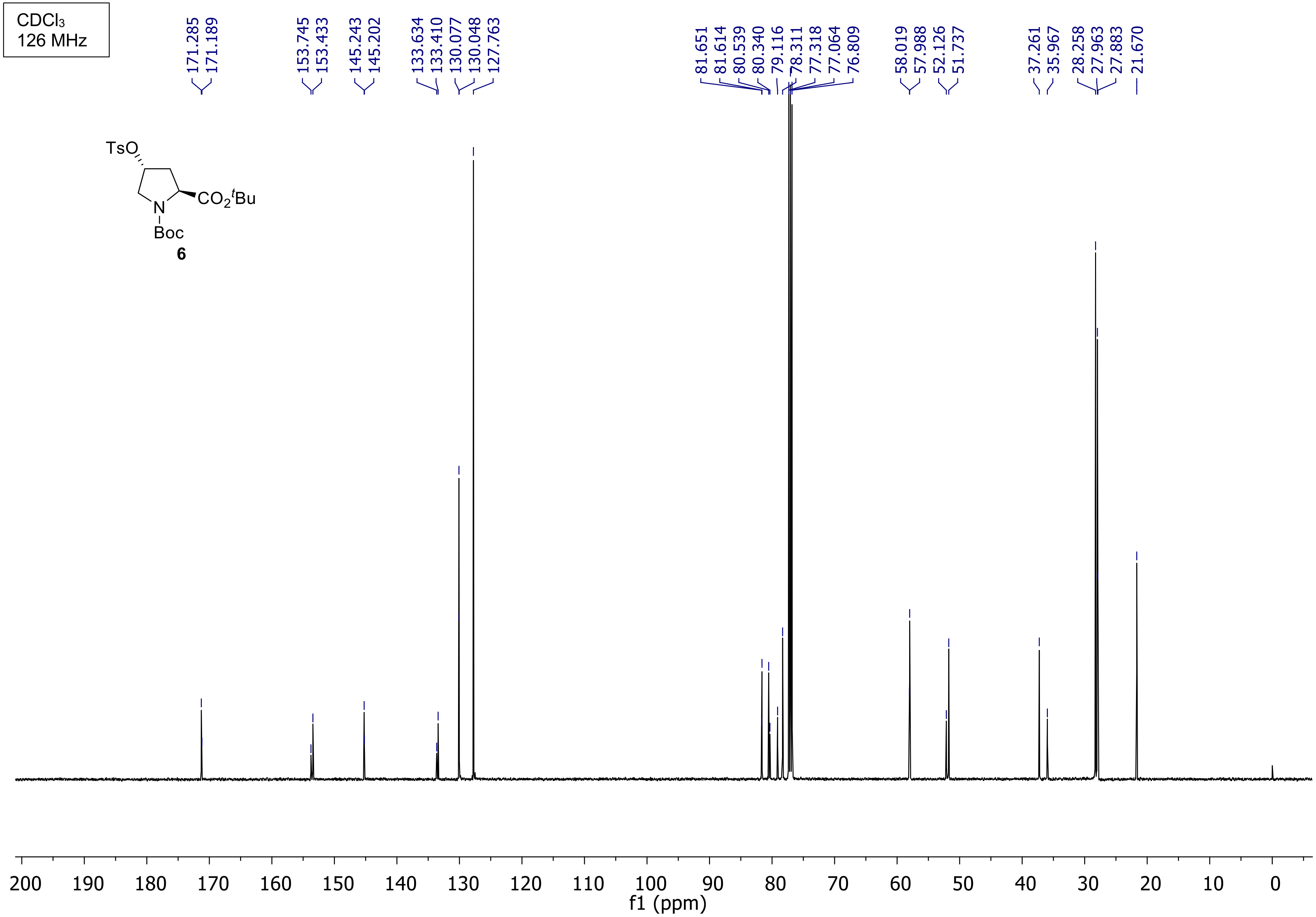
CDCl3
126 MHz
171.285
171.189
153.745
153.433
145.243
145.202
133.634
133.410
130.077
130.048
127.763
81.651
81.614
80.539
80.340
79.116
78.311
77.318
77.064
76.809
58.019
57.988
52.126
51.737
37.261
35.967
28.258
27.963
27.883
21.670
TsO
CO2tBu
N
Boc
6
200 190 180 170 160 150 140 130 120 110 100 90 80 70 60 50 40 30 20 10 0
f1 (ppm)

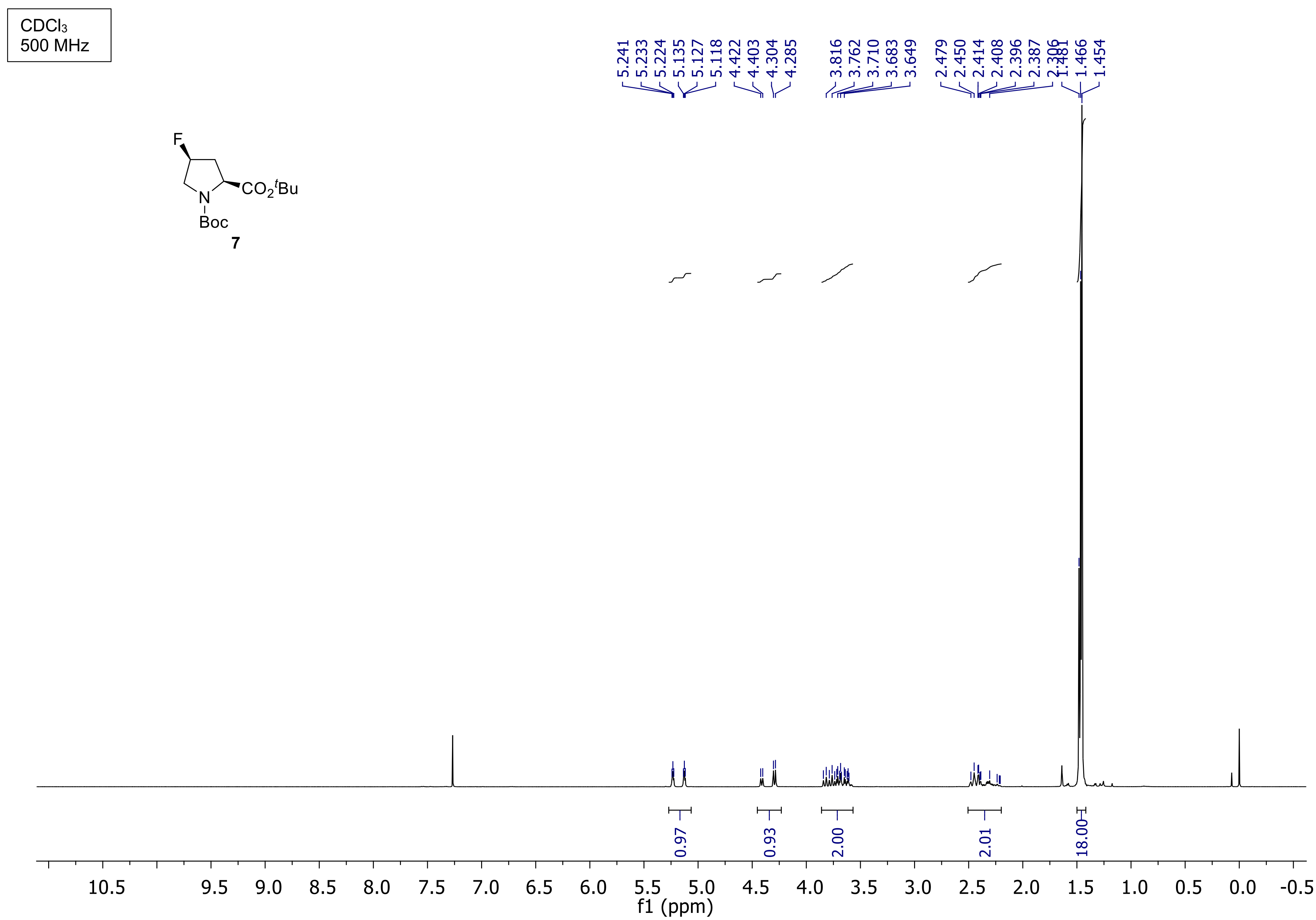
CDCl3
500 MHz
F
CO2tBu
N
Boc
7
5.241
5.233
5.224
5.135
5.127
5.118
4.422
4.403
4.304
4.285
3.816
3.762
3.710
3.683
3.649
2.479
2.450
2.414
2.408
2.396
2.387
2.306
1.481
1.466
1.454
0.97
0.93
2.00
2.01
18.00
10.5
9.5
9.0
8.5
8.0
7.5
7.0
6.5
6.0
5.5
5.0
4.5
4.0
3.5
3.0
2.5
2.0
1.5
1.0
0.5
0.0
-0.5
f1 (ppm)

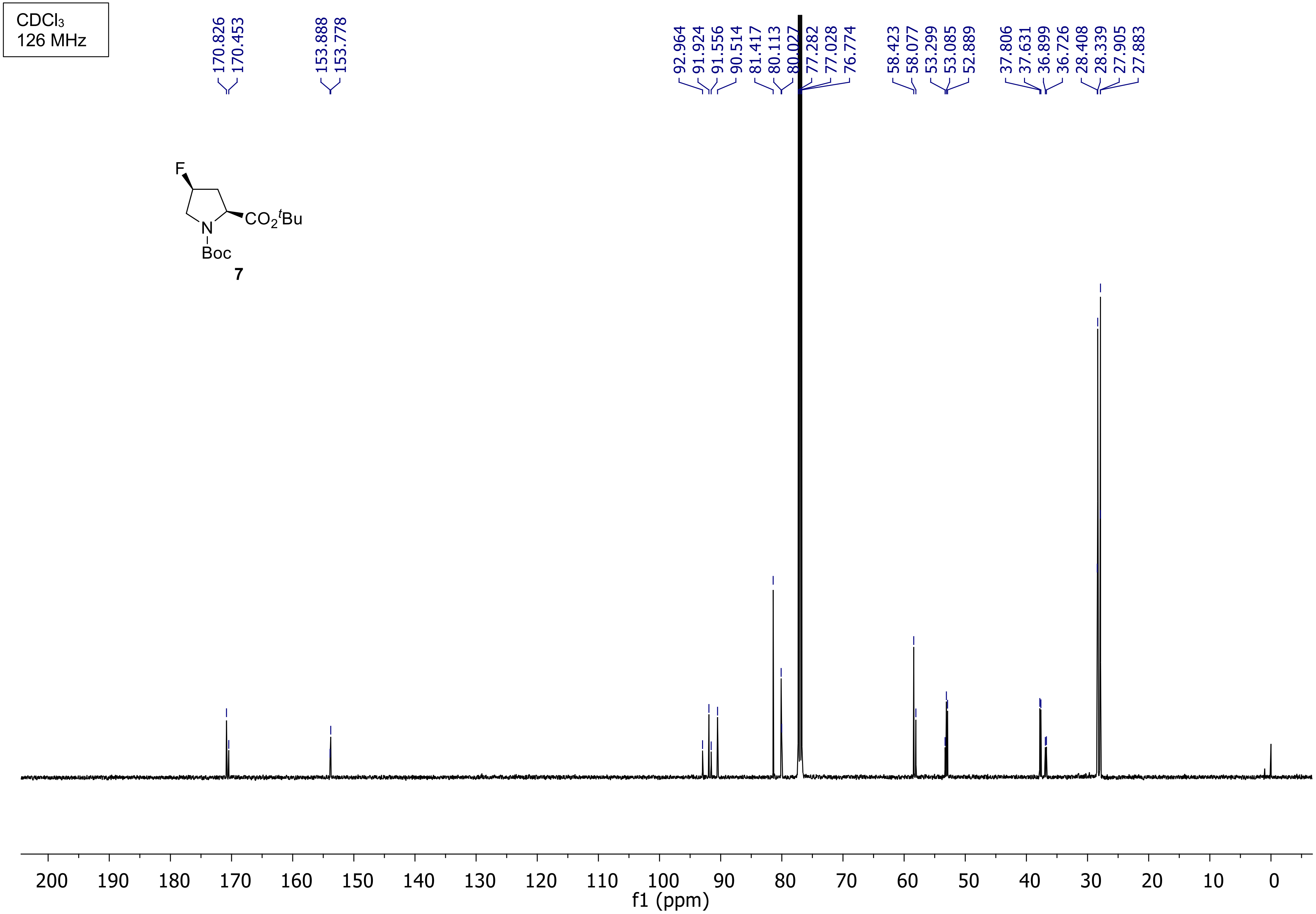
CDCl3
126 MHz
170.826
170.453
153.888
153.778
92.964
91.924
91.556
90.514
81.417
80.113
80.027
77.282
77.028
76.774
58.423
58.077
53.299
53.085
52.889
37.806
37.631
36.899
36.726
28.408
28.339
27.905
27.883
F
CO2tBu
N
Boc
7
200 190 180 170 160 150 140 130 120 110 100 90 80 70 60 50 40 30 20 10 0
f1 (ppm)

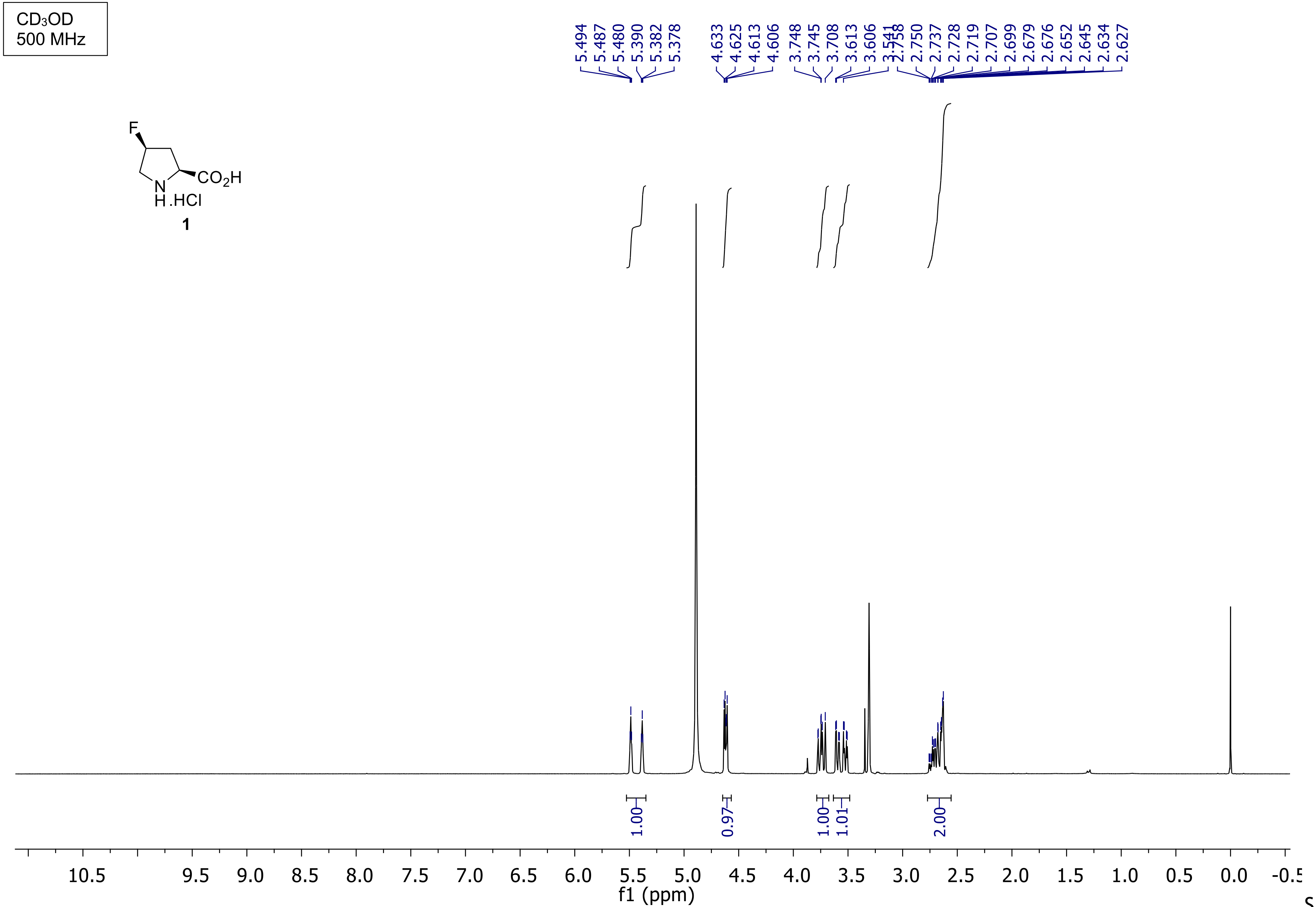

CD3OD
500 MHz
F
CO2H
N
H.HCl
1
5.494
5.487
5.480
5.390
5.382
5.378
4.633
4.625
4.613
4.606
3.748
3.745
3.708
3.613
3.606
3.541
2.758
2.750
2.737
2.728
2.719
2.707
2.699
2.679
2.676
2.652
2.645
2.634
2.627
1.00
0.97
1.00
1.01
2.00
10.5
9.5
9.0
8.5
8.0
7.5
7.0
6.5
6.0
5.5
5.0
4.5
4.0
3.5
3.0
2.5
2.0
1.5
1.0
0.5
0.0
-0.5
f1 (ppm)

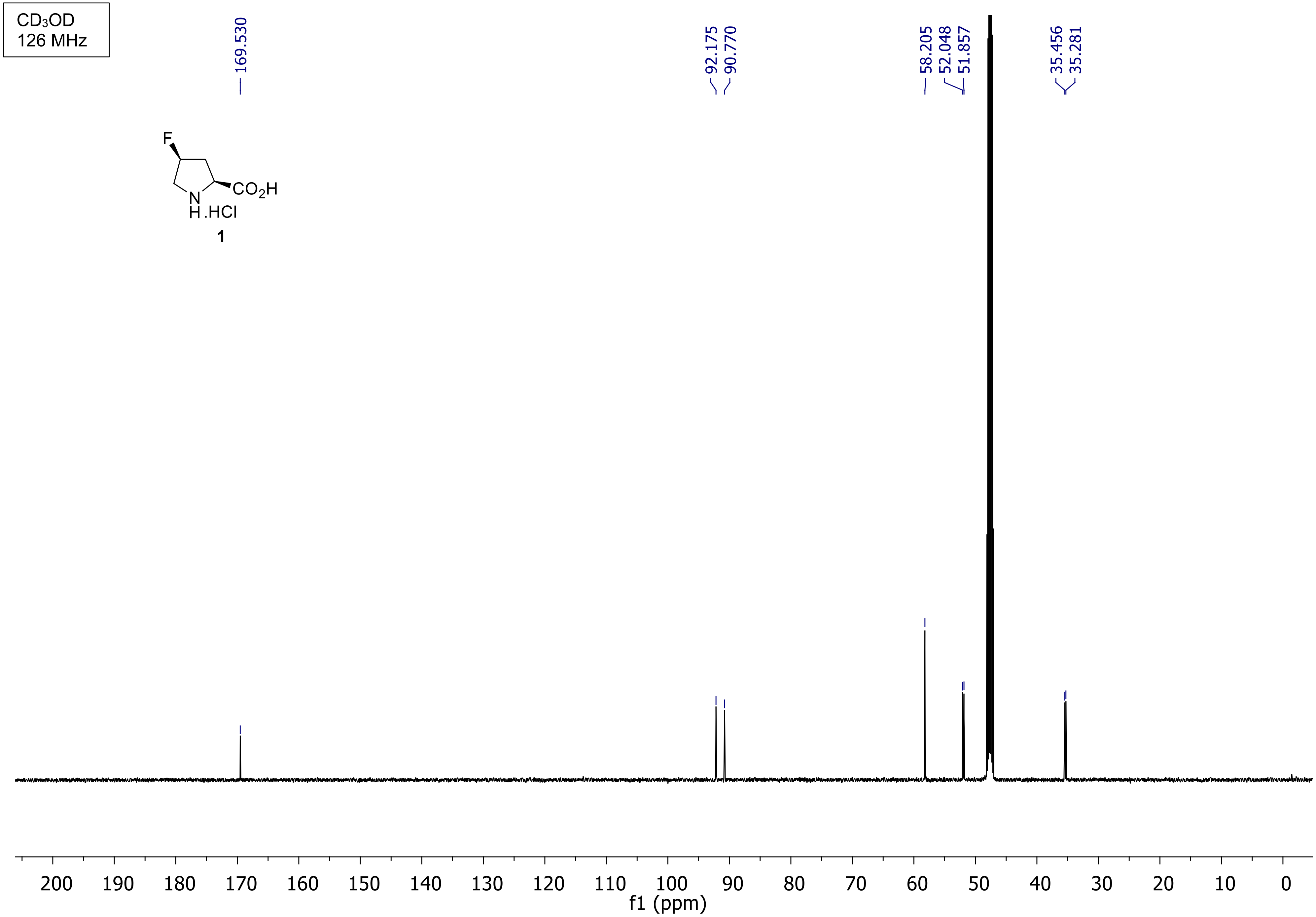

CD3OD
126 MHz
169.530
92.175
90.770
58.205
52.048
51.857
35.456
35.281
F
CO2H
N
H .HCl
1
200
190
180
170
160
150
140
130
120
110
100
90
80
70
60
50
40
30
20
10
0
f1 (ppm)

CDCl$_3$
400 MHz

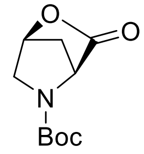

8

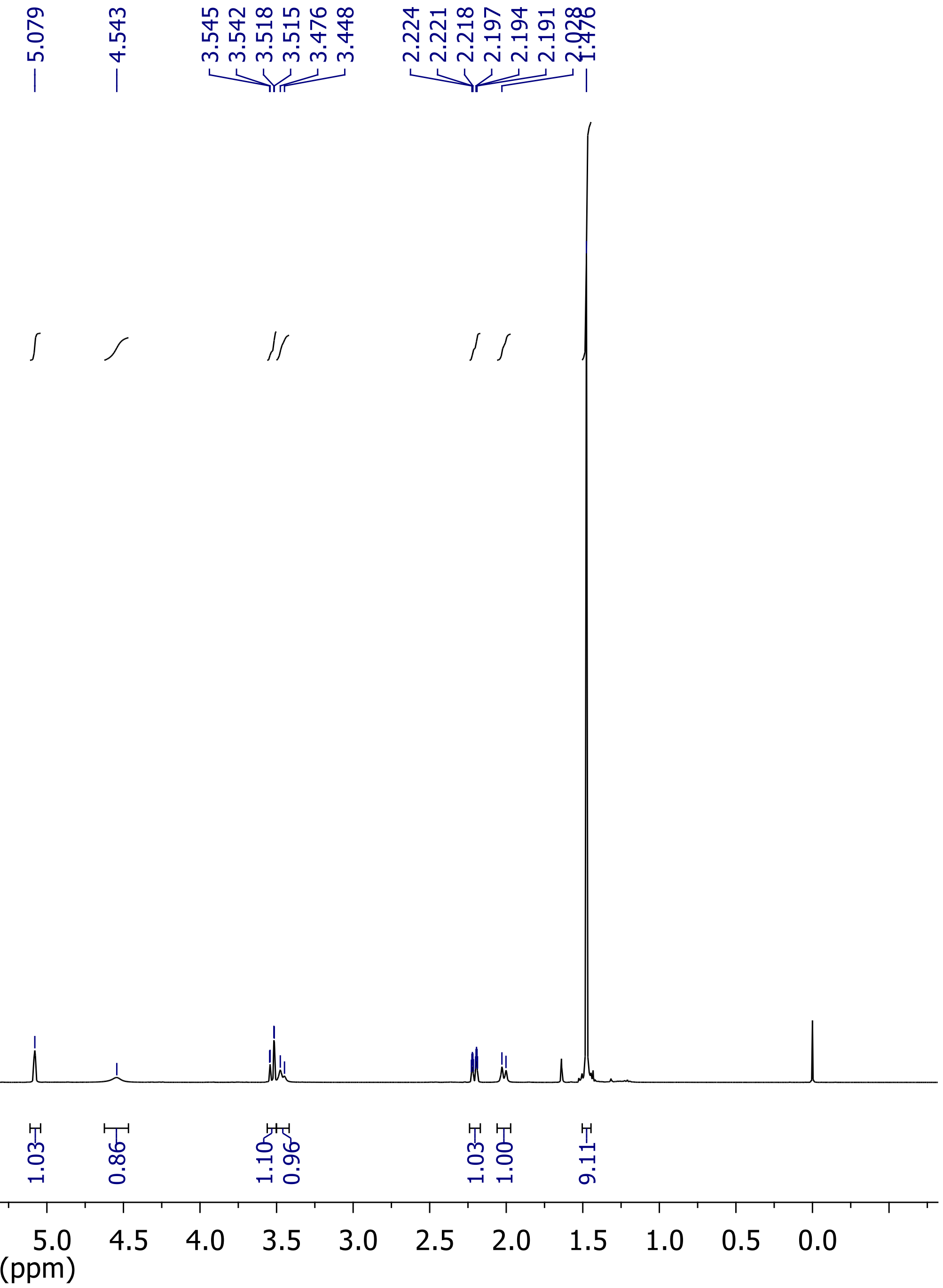

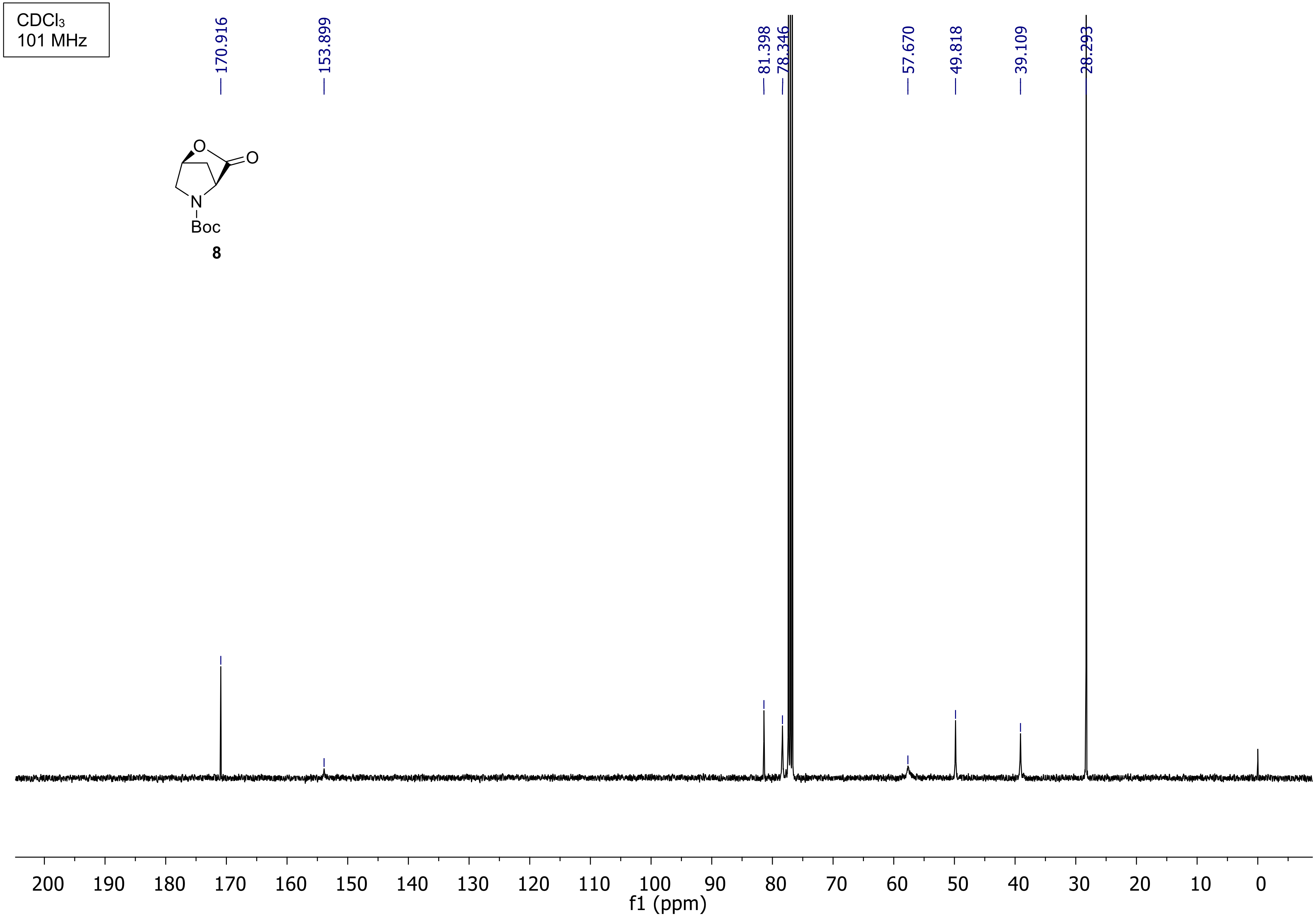
CDCl3
101 MHz
170.916
153.899
81.398
78.346
57.670
49.818
39.109
28.293
O
O
N
Boc
8
200 190 180 170 160 150 140 130 120 110 100 90 80 70 60 50 40 30 20 10 0
f1 (ppm)

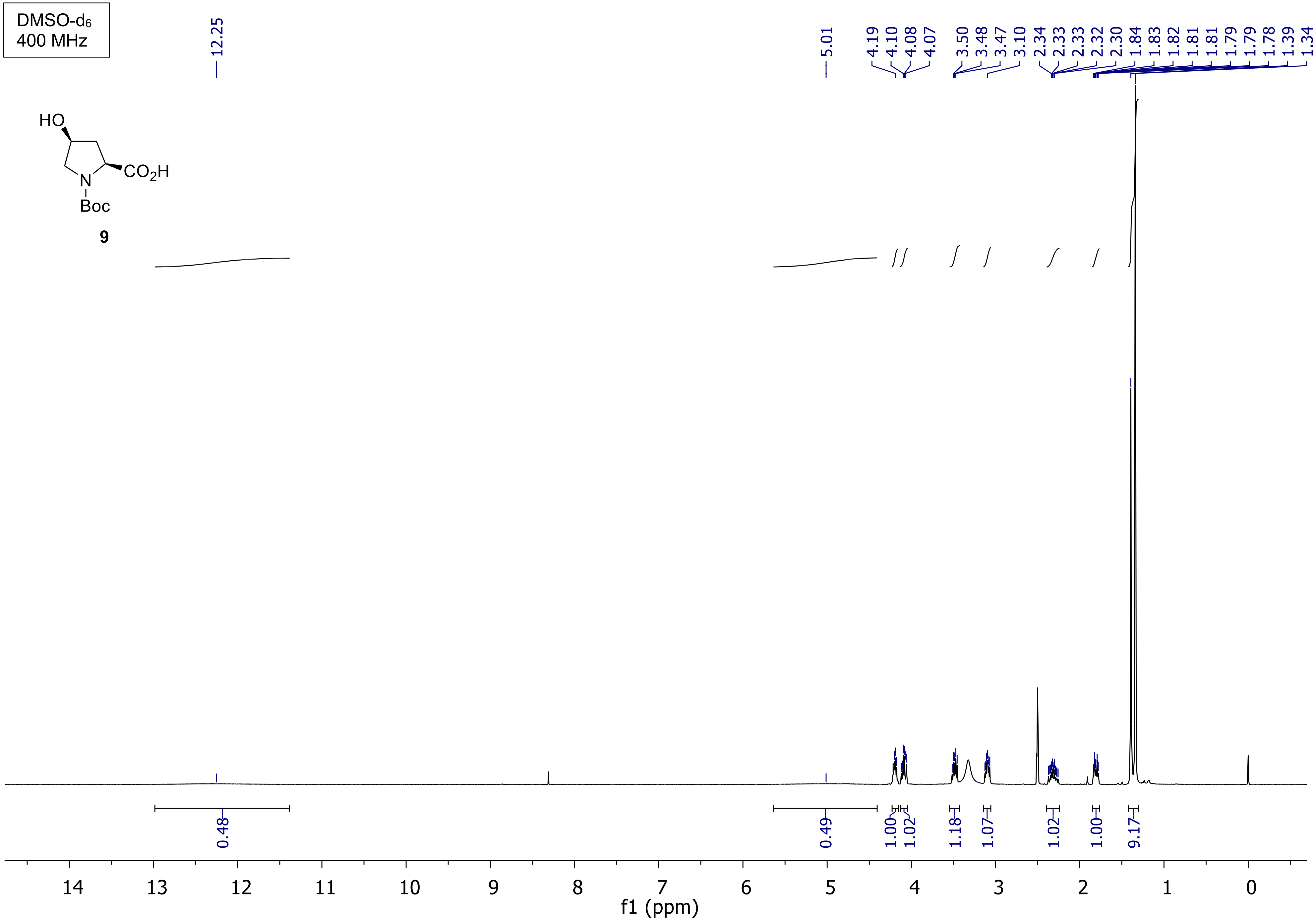
DMSO-d6
400 MHz
HO
CO2H
N
Boc
9
12.25
5.01
4.19
4.10
4.08
4.07
3.50
3.48
3.47
3.10
2.34
2.33
2.33
2.32
2.30
1.84
1.83
1.82
1.81
1.81
1.79
1.79
1.78
1.39
1.34
0.48
0.49
1.00
1.02
1.18
1.07
1.02
1.00
9.17
14
13
12
11
10
9
8
7
6
5
4
3
2
1
0
f1 (ppm)

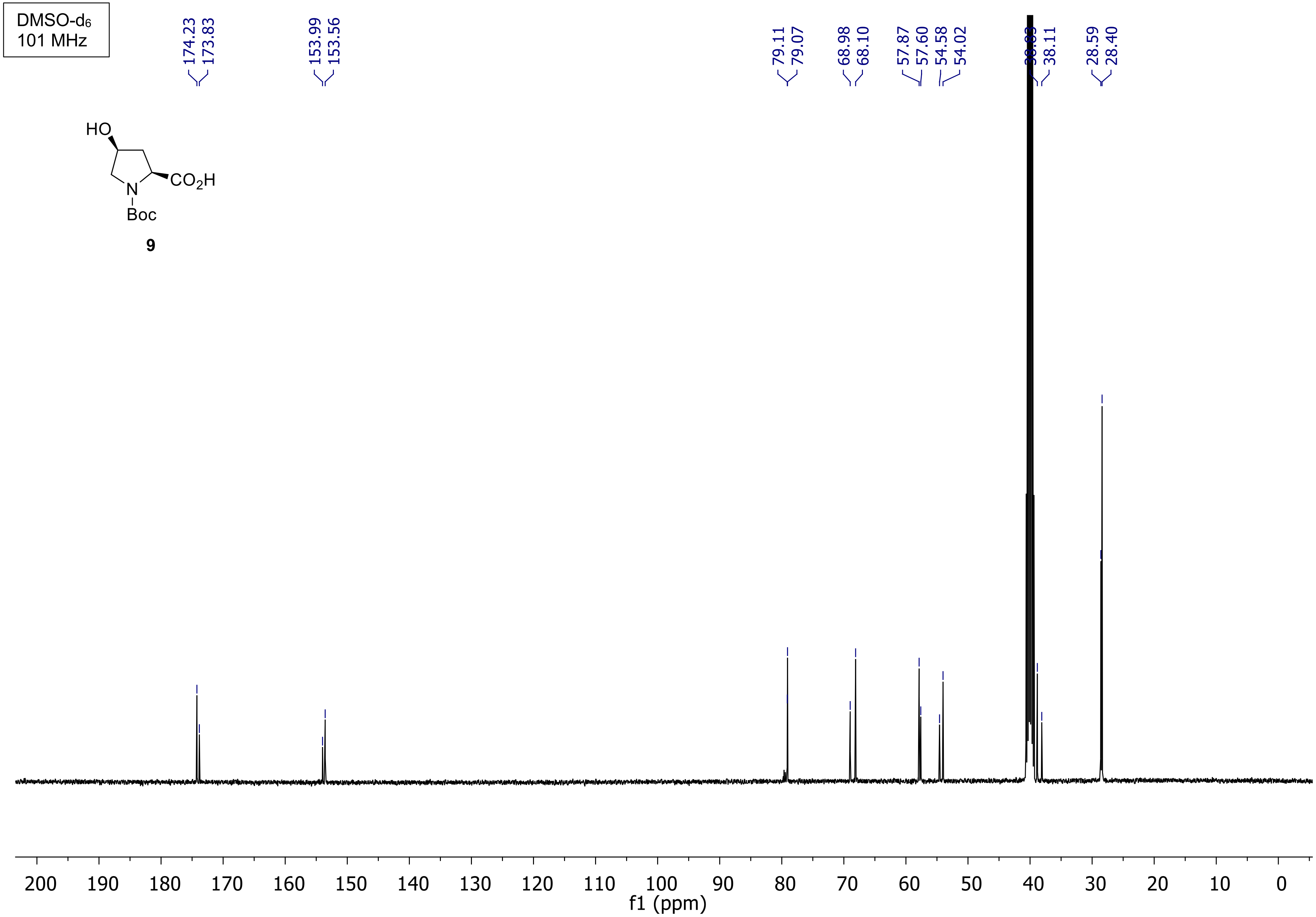
DMSO-d6
101 MHz
174.23
173.83
153.99
153.56
79.11
79.07
68.98
68.10
57.87
57.60
54.58
54.02
38.11
28.59
28.40
HO
CO_2H
N
Boc
9
200 190 180 170 160 150 140 130 120 110 100 90 80 70 60 50 40 30 20 10 0
f1 (ppm)

CDCl3
400 MHz

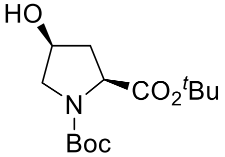

10

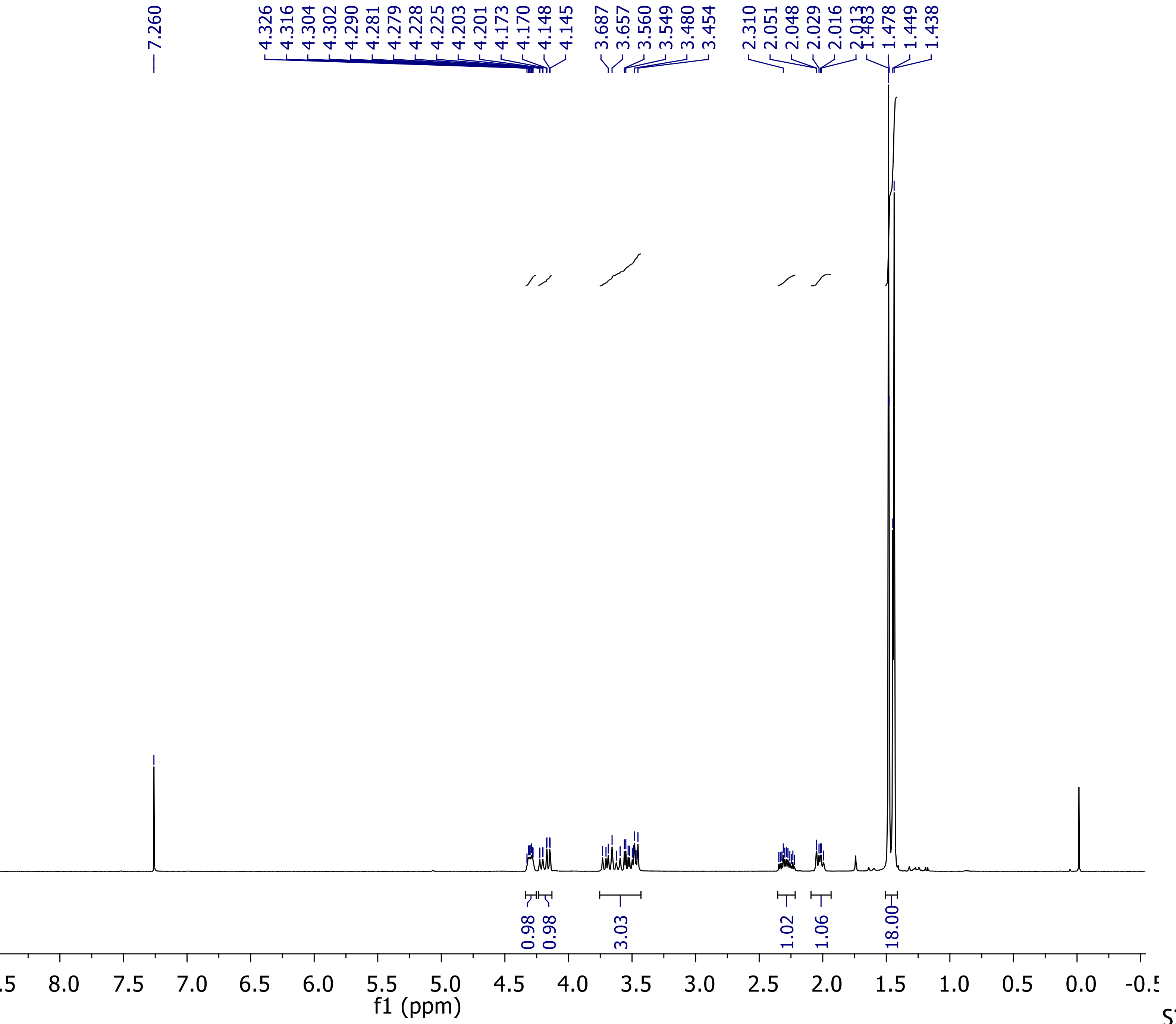

CDCl3
101 MHz

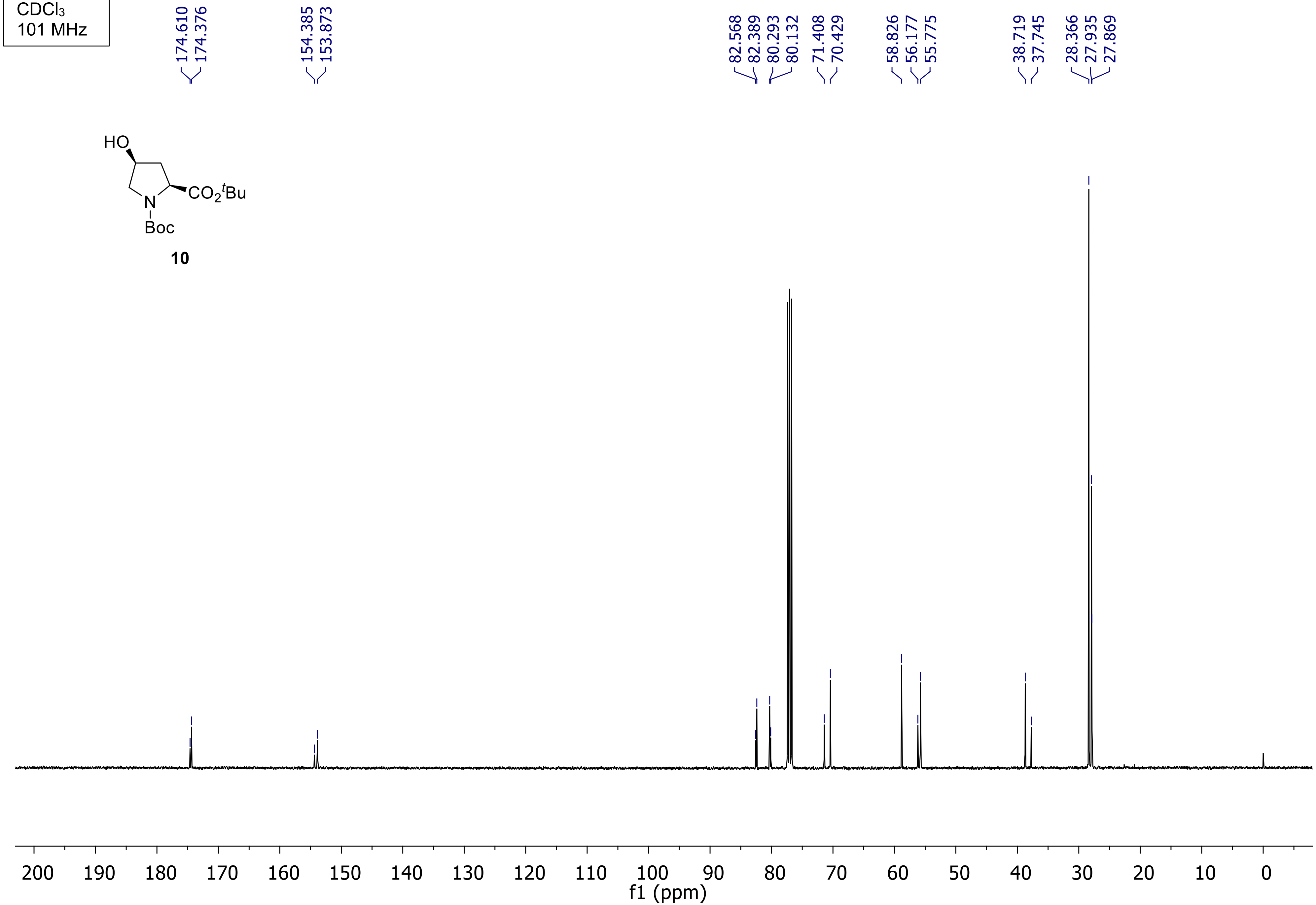

CDCl3
400 MHz

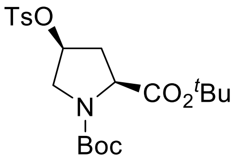

11

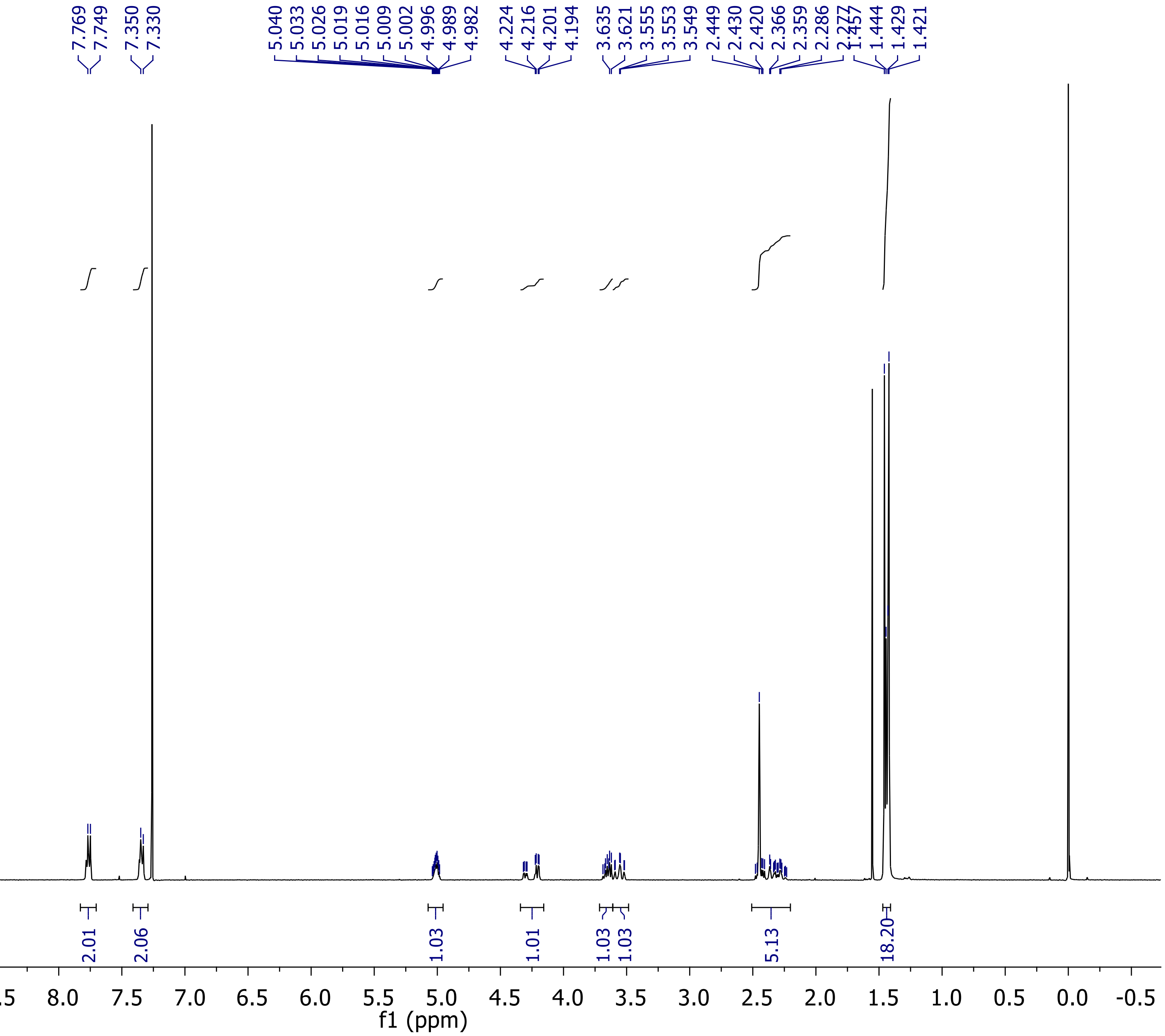

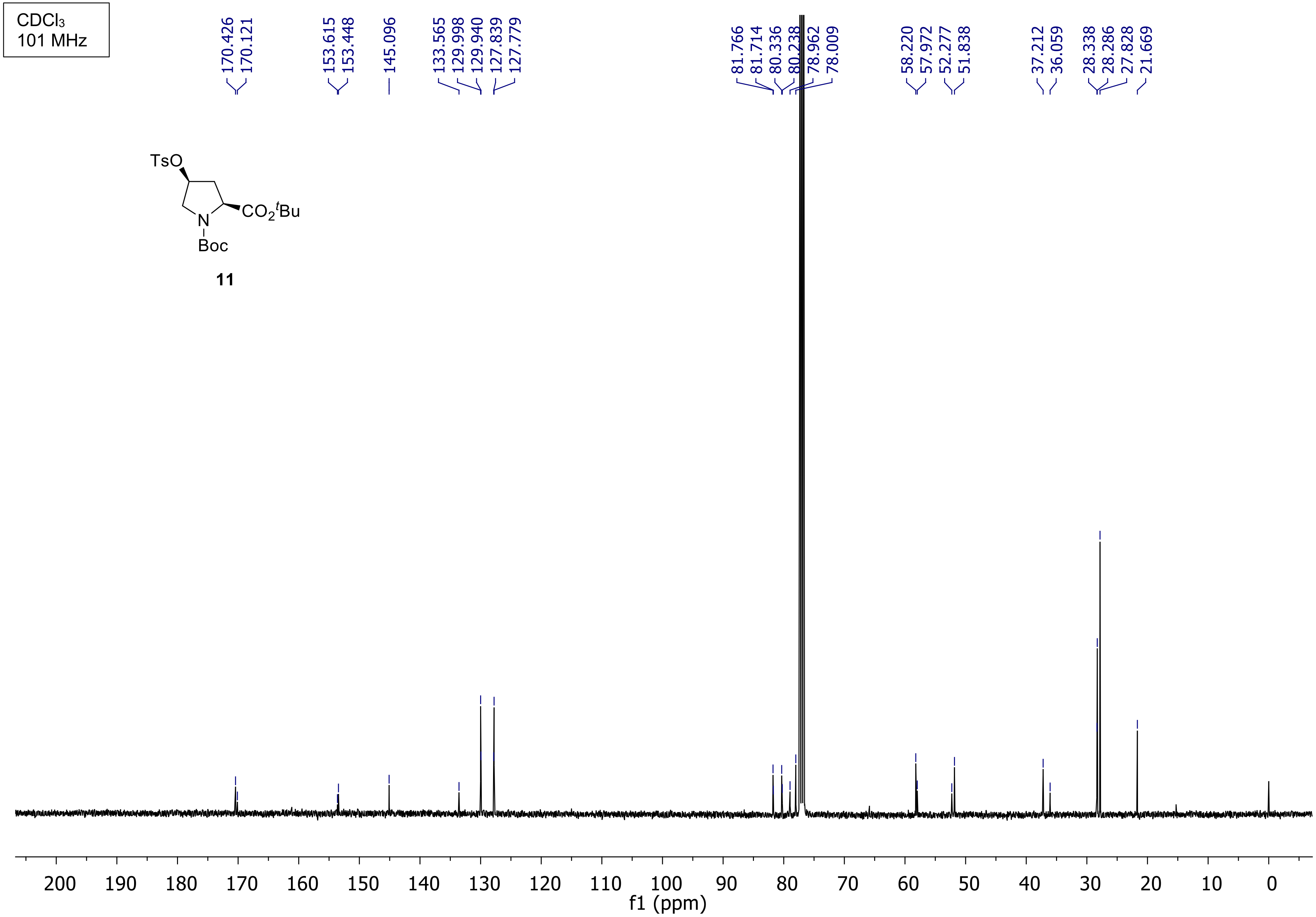

CDCl3
101 MHz
170.426
170.121
153.615
153.448
145.096
133.565
129.998
129.940
127.839
127.779
81.766
81.714
80.336
80.238
78.962
78.009
58.220
57.972
52.277
51.838
37.212
36.059
28.338
28.286
27.828
21.669
TsO
CO2tBu
N
Boc
200 190 180 170 160 150 140 130 120 110 100 90 80 70 60 50 40 30 20 10 0
f1 (ppm)

11

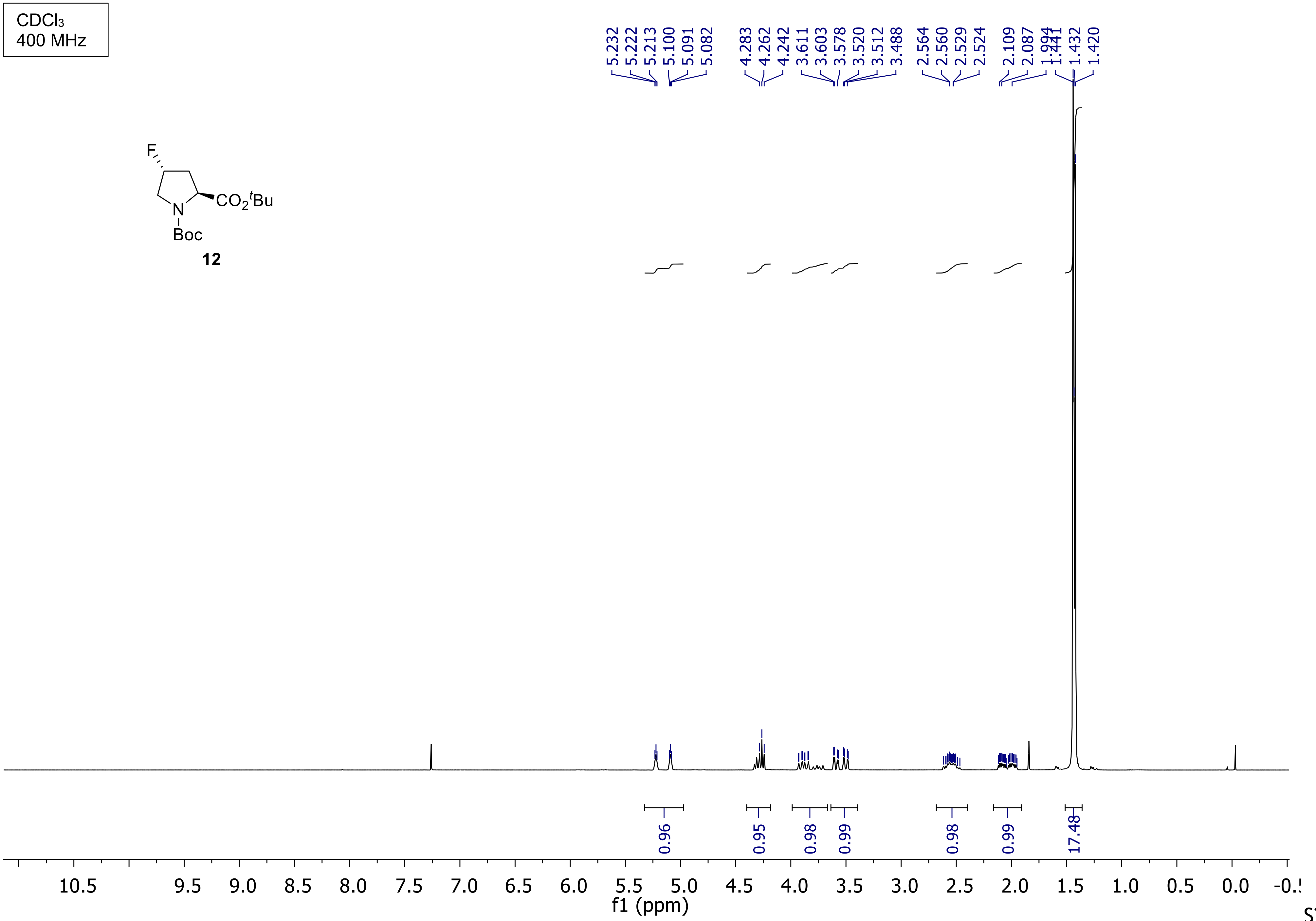
CDCl3
400 MHz
F
N
CO2tBu
Boc
12
5.232
5.222
5.213
5.100
5.091
5.082
4.283
4.262
4.242
3.611
3.603
3.578
3.520
3.512
3.488
2.564
2.560
2.529
2.524
2.109
2.087
1.994
1.441
1.432
1.420
0.96
0.95
0.98
0.99
0.98
0.99
17.48
10.5
9.5
9.0
8.5
8.0
7.5
7.0
6.5
6.0
5.5
5.0
4.5
4.0
3.5
3.0
2.5
2.0
1.5
1.0
0.5
0.0
f1 (ppm)

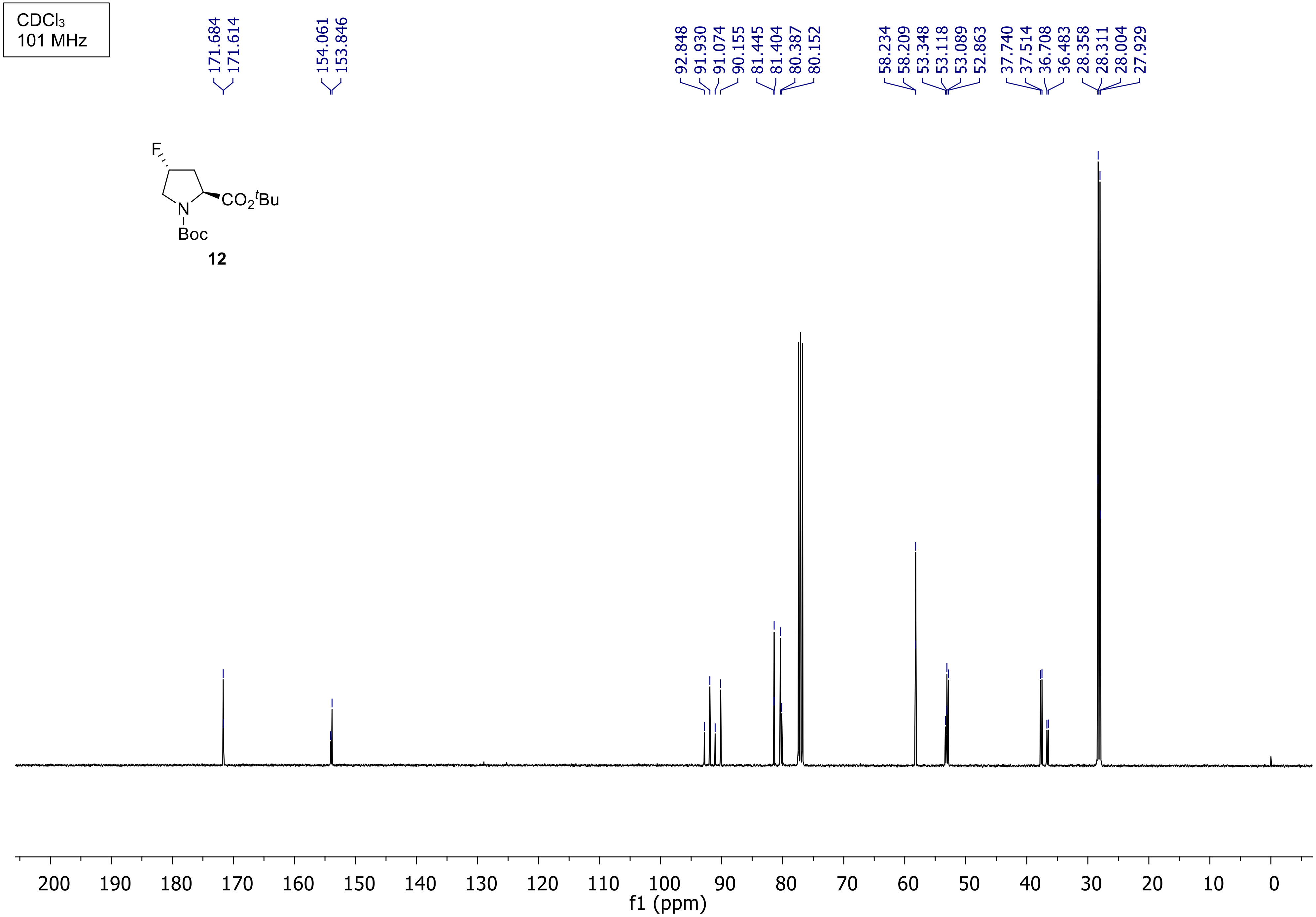
CDCl3
101 MHz
171.684
171.614
154.061
153.846
92.848
91.930
91.074
90.155
81.445
81.404
80.387
80.152
58.234
58.209
53.348
53.118
53.089
52.863
37.740
37.514
36.708
36.483
28.358
28.311
28.004
27.929
F
CO2tBu
N
Boc
12
200 190 180 170 160 150 140 130 120 110 100 90 80 70 60 50 40 30 20 10 0
f1 (ppm)

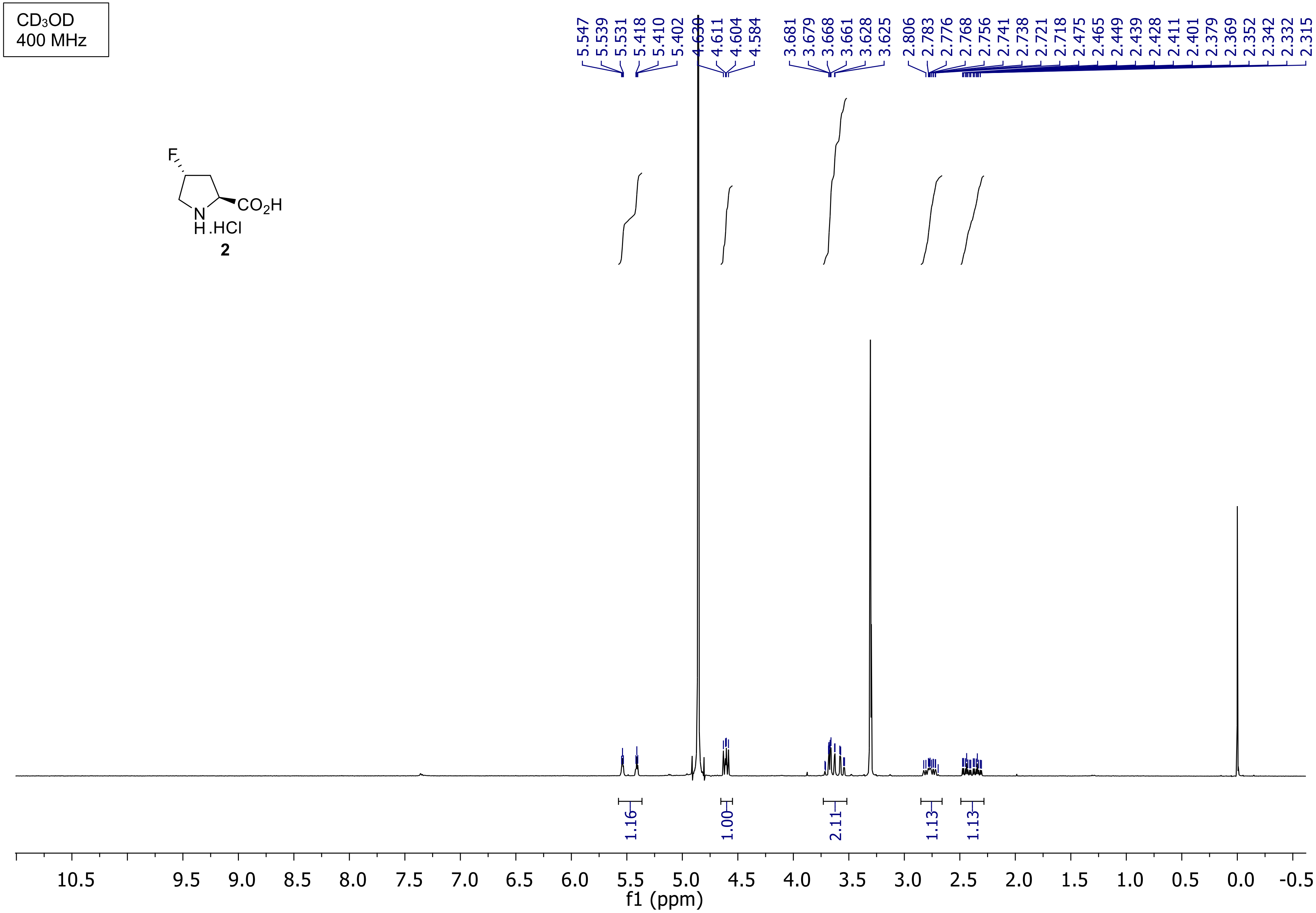

CD3OD
400 MHz
F
CO2H
N
H .HCl
2
5.547
5.539
5.531
5.418
5.410
5.402
4.630
4.611
4.604
4.584
3.681
3.679
3.668
3.661
3.628
3.625
2.806
2.783
2.776
2.768
2.756
2.741
2.738
2.721
2.718
2.475
2.465
2.449
2.439
2.428
2.411
2.401
2.379
2.369
2.352
2.342
2.332
2.315
1.16
1.00
2.11
1.13
1.13
10.5
9.5
9.0
8.5
8.0
7.5
7.0
6.5
6.0
5.5
5.0
4.5
4.0
3.5
3.0
2.5
2.0
1.5
1.0
0.5
0.0
-0.5
f1 (ppm)

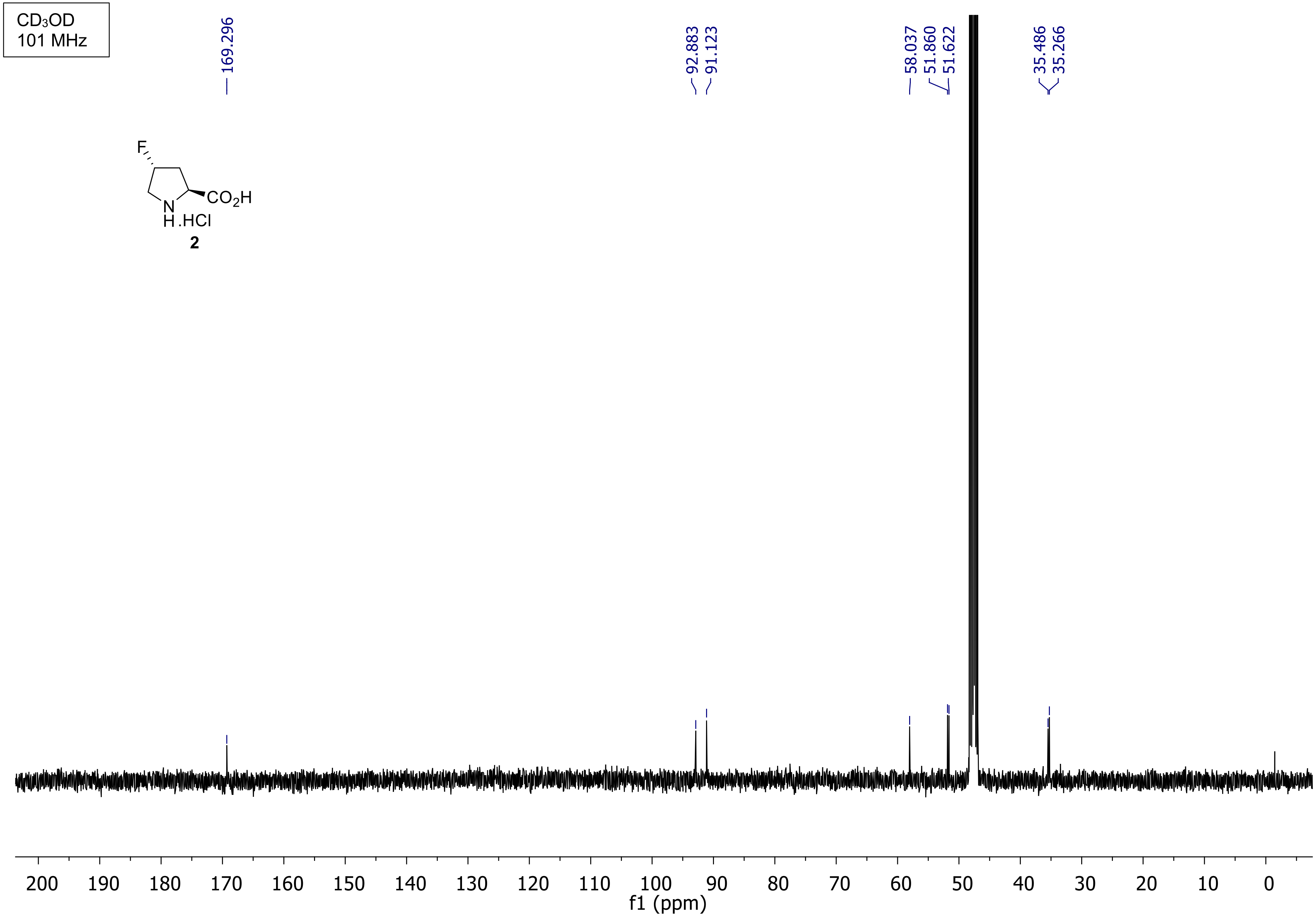
CD3OD
101 MHz
169.296
92.883
91.123
58.037
51.860
51.622
35.486
35.266
F
N
H.HCl
CO2H
2
200 190 180 170 160 150 140 130 120 110 100 90 80 70 60 50 40 30 20 10 0
f1 (ppm)
